# Decorin Deficiency Promotes D‐Galactose–Induced Skeletal Muscle Atrophy and Fibrosis by Regulating ITGB1/Akt/mTOR Signalling Pathway

**DOI:** 10.1002/jcsm.70144

**Published:** 2025-12-05

**Authors:** Xiaoqin Luo, Mengxue Zhang, Lili Chen, Ruichang Wang, Jiabing Lian, Zhe Yang, Renato V. Iozzo, Jin Wang, Yan Zhang, Xiuli Bi

**Affiliations:** ^1^ College of Life Science Liaoning University Shenyang China; ^2^ Department of Pathology, Anatomy and Cell Biology, and Cancer Cell Biology and Signaling Program, Sidney Kimmel Cancer Center Thomas Jefferson University Philadelphia Pennsylvania USA; ^3^ Spine Disease Research Institute, Longhua Hospital Shanghai University of Traditional Chinese Medicine Shanghai China; ^4^ Key Laboratory of Theory and Therapy of Muscles and Bones Ministry of Education Shanghai China; ^5^ Key Laboratory for Chronic Diseases Molecular Mechanism Research and Nutritional Intervention of Shenyang Shenyang China

**Keywords:** Akt/mTOR, decorin, fibrosis, sarcopenia, skeletal muscle atrophy

## Abstract

**Background:**

Primary sarcopenia is an age‐associated disorder with progressive and generalised loss of skeletal muscle strength and mass. Skeletal muscle fibrosis is one of the significant pathological manifestations of age‐associated sarcopenia. Decorin, a small dermatan–sulfate proteoglycan, participates in extracellular matrix assembly. In numerous studies, the involvement of decorin is not restricted to matrix structural proteins, and it also affects a diverse variety of biological functions like cell growth, adhesion, migration, proliferation and differentiation. Additionally, it modulates the process of inflammation and fibrillogenesis. Based on these preclinical evidences, we hypothesised that decorin may potentially play an important role in skeletal muscles during ageing.

**Methods:**

Natural ageing mice (Dcn^+/+^), D‐galactose (D‐gal)–induced Dcn^+/+^, Dcn^−/−^ mice and NOR‐10 cell models were established. Grip strength and exercise capacity were evaluated, after the mice were sacrificed to collect their gastrocnemius muscles for assessment of atrophy and fibrosis by haematoxylin and eosin staining, qRT‐PCR and Western blotting. Co‐immunoprecipitation was used to identify the interaction between decorin and integrin β1 (ITGB1).

**Results:**

The expression levels of decorin were reduced at both mRNA and protein levels in muscle during natural ageing in mice (−75.3% of mRNA level; −29.5% of protein level) and NOR‐10 cells (−78% of protein level). *si‐Dcn* promoted the expression of α‐SMA (+37.2%) and fibronectin (+53.1%), which are related to muscle fibrosis in D‐gal–induced NOR‐10 cells. In addition, in Dcn^−/−^–D‐gal mice, which exhibited more aggravated muscle atrophy including smaller grip strength (−21.7%, *p* < 0.001), downregulation of the ratio of gastrocnemius weight (−7.3%), fibre size (Gast: −15.3%) and increased levels of α‐SMA (+70.2%), MuRF‐1 (+30.2%), NLRP3(+19.4%) and p21(+27.2%) proteins compared with Dcn^+/+^–D‐gal mice. In terms of the underlying mechanisms, the Akt/mTOR signalling pathway was downregulated in Dcn^−/−^–D‐gal mice compared with aged Dcn^+/+^–D‐gal mice (p‐S473‐Akt/Akt: −38.1%; p‐Ser2448‐mTOR/mTOR: −28.8%; p‐p70S6K/p70: −40.3%; p‐4E‐BP1: −42.3%, *p* < 0.05). Decorin activation enhanced ITGB1 expression (+46.7% vs. NC, *p* < 0.01). si‐ITGB1 suppressed the expression of p‐S473‐Akt and p‐Ser2448‐mTOR in NOR‐10 cells with Dcn overexpression. The interaction between decorin and ITGB1 was found in skeletal muscle, suggesting that the regulation of decorin on Akt/mTOR might depend on ITGB1. Notably, decorin deficiency increased the accumulation of p62 (+47.2%, *p* < 0.05) and LC3b (+50.9%, *p* < 0.01).

**Conclusions:**

The comprehensive results show that decorin plays a sarcoprotective role by activating the ITGB1/Akt/mTOR pathway and may serve as a potential therapeutic reagent in age‐associated sarcopenia.

## 1 Introduction

Ageing constitutes the biggest risk factor for poor health and adversely affects the integrity and function of all the cells, tissues and organs in the human body [1]. During ageing, skeletal muscle undergoes functional decline, leading to age‐associated sarcopenia, which is characterised by loss of skeletal muscle mass and impaired function [2, 3, 4]. Age‐associated sarcopenia imposes a significant economic burden on both society and the healthcare system. Therefore, there is an urgent need to investigate the potential mechanisms and effective intervention targets for sarcopenia.

A notable feature of senescent tissue and pathological skeletal muscle is the development of fibrosis [5]. There is a strong relationship between increased muscle fibrosis‐related factors and loss of muscle strength, which fibrosis impairs muscle function hindering muscle regeneration after injury [6]. For example, the expression levels of TGF‐β1 and α‐SMA were significantly higher in aged mice compared to young mice, indicating that skeletal muscle in aged mice exhibits significant fibrosis [7]. In addition, interstitial fibrosis is clearly observed in the skeletal muscle of older adults and the increase in fibrous tissue leads to muscle stiffness, limiting muscle extension and contraction, thus reducing exercise capacity in older adults [8, 9]. Although these studies have confirmed the importance of skeletal muscle fibrosis in the pathogenesis of age‐associated sarcopenia, the molecular mechanisms underlying muscle fibrosis have not yet been fully elucidated.

Integrins, which consist of two non‐covalently linked α and β subunits, play a crucial role in cell–cell adhesion and cell‐extracellular matrix (ECM) interactions [10, 11]. Among them, integrin β1 (ITGB1) is the most common subunit, which plays a role in several steps of myogenesis, including migration, differentiation and fusion [12, 13]. The interaction between ITGB1 and ECM activates ITGB1‐mediated intracellular signalling pathways, such as focal adhesion kinase (FAK) pathway, mitogen‐activated protein kinase (MAPK) pathway and phosphoinositide 3‐kinase (PI3K)/protein kinase B (Akt) pathway [14]. These ITGB1‐mediated downstream pathways have been implicated in myogenesis and skeletal muscle regeneration [15]. Meanwhile, muscle mass is maintained through the regulation of anabolic pathways. The most significant pathway is the PI3K/Akt/mammalian target of rapamycin (mTOR) pathway, which promotes skeletal muscle differentiation, induces protein synthesis and blocks degradation [16, 17, 18]. Akt activates mTOR to induce protein synthesis by regulating the ribosomal protein S6 kinase beta‐1/p70S6 kinase (p70S6K) and the eukaryotic translation initiation factor 4E (eIF4E)‐binding protein 1 (4EBP1) [19]. In response to chronic low‐grade inflammation, inflammatory factors such as TNF‐α and IL‐6 can reach an aberrant level and inhibit the activity of the PI3K/AKT/mTOR pathway, which contributes to reduced muscle protein synthesis and increased degradation of muscle protein [20].

Decorin was identified as a myokine, which is an important component of the ECM. Decorin is widely distributed in mesenchymal tissues and expressed and secreted in a variety of connective tissues [21, 22]. It was revealed to inhibit migration, invasion and epithelial–mesenchymal transition (EMT) by suppressing the c‐mesenchymal–epithelial transition factor (c‐Met)/Akt/mTOR signalling pathway [23]. Decorin deficiency causes collagen deposition during corneal wound healing in mice [24]. A clinical cohort study showed that myostatin increased and decorin reduced with inflammation, hyperammonaemia and malnutrition in alcohol‐associated liver disease [25]. In another study, high expression of decorin ameliorates TGF‐β‐induced fibrosis in a recessive dystrophic epidermolysis bullosa mice model (RDEB) [26]. However, whether and how decorin is involved in the progress of sarcopenia is unknown. In the present study, we delved into evaluating whether decorin could effectively mitigate fibrosis in ageing skeletal muscle, thereby attenuating muscle atrophy and the underlying mechanism. These findings may shed light on the discovery of a promising therapeutic reagent for sarcopenia treatment.

## 2 Materials and Methods

### 2.1 Animals

All experimental procedures in this study were approved by the Animal Ethics Committee of Liaoning University. Procedures for animal handling and tissue sampling were conducted in compliance with protocols approved by the Animal Care and Use Committee of Liaoning University. Mice were maintained on a 12‐h light/dark cycle and housed in an individually ventilated cage facility with ad libitum access to food and water. A standard chow diet (AIN93G) and water were provided ad libitum during the experiments. In this study, wild‐type C57BL/6 J (Dcn^+/+^) mice and C57BL/6 J decorin‐deficient (Dcn^−/−^) mice were generated from a breeding pair of Dcn^+/−^ mice kindly provided by Professor Renato Iozzo, Sidney Kimmel Medical College, Thomas Jefferson University, Philadelphia, PA [27]. Dcn^+/+^ and Dcn^−/−^ male mice with an age of 6–8 weeks were selected for experiments after genotyping through genomic DNA extraction from tail tissue (Figure S2a). For natural ageing mice, the 3‐ and 18‐month‐old Dcn^+/+^ male mice were used for the comparison of young and aged mice (*n* = 6).

### 2.2 D‐Gal–Induced Sarcopenia Mice Model

For the D‐galactose (D‐gal)–induced sarcopenia mice model experiments, the 8‐week‐old male C57BL/6 J mice (20 ± 2 g) were randomly divided into three groups as follows: (1) The control group mice were injected intraperitoneally with saline for 10 weeks (Dcn^+/+^ group, *n* = 6). (2) The Dcn^+/+^–D‐gal and Dcn^−/−^–D‐gal group mice were treated with D‐gal (500 mg/kg) once a day for 10 weeks (Dcn^+/+^–D‐gal group, *n* = 6; Dcn^−/−^–D‐gal group, *n* = 6). After 10 weeks, blood samples were collected from the tail vein and centrifuged at 3000 rpm for 20 min to separate the serum. During the experiment, the general conditions of mice in each group were observed weekly, including food intake, hair removal, weakness, physical activity etc.

### 2.3 Analysis of Physical Performance Tests

The age‐dependent physical performance of C57BL/6 J mice was tested, including grip strength and wire hang. We used forearm grip strength to measure muscle strength in the grip strength test, and suspension force was tested by the wire hang test. During the grip strength test, each mousegrabbed a grip meter with its forelimb and gradually pulled back until it released its grip. The maximum force was recorded in Newton (N). The tests were repeated five times. The maximum among the five times was recorded as the grip strength of individual mice. In the wire hang test, the mice were placed onto the top of a wire mesh cage, which was then gently inverted to encourage the mice to grip the wire; the retention time was recorded. We set the maximum duration at 2 min. The average of the two times was recorded as the individual mice muscle strength.

### 2.4 Muscle Fatigue Test

Muscular endurance test was performed by the motorised treadmill. After 3 days of acclimation, the mice were placed on the treadmill with the speed of 6 m/min. The running speed were increased every 5 min with 1 m/min until the mice showed fatigue defined by an inability to return to the treadmill or staying on the electrical shock grids for 10 s.

### 2.5 Tissue Collection

After the examination, the mice were sacrificed by dislocation of cervical vertebra. After the mice were killed, the muscle was separated immediately. The gastrocnemius (Gast), tibialis anterior (TA) and quadriceps (QU) were excised from both hindlimbs. The gastrocnemius from left leg was fixed in 4% paraformaldehyde for histological staining, and the other muscles of left leg were frozen in liquid nitrogen immediately. The muscles from the right leg were weighed and frozen in liquid nitrogen. All tissue samples were stored at −80°C until analysis. A part of these tissues was used for Western blotting analysis.

### 2.6 Haematoxylin–Eosin Staining

For histological analyses, isolated gastrocnemius tissues were pre‐washed with normal saline and fixed for at least 24 h in 4% paraformaldehyde. After immersion fixation and dehydration embedding, paraffin sections (5 μm) of the muscles were prepared on a microtome (Leica RM2235). Paraffin sections of skeletal muscles were stained with haematoxylin and eosin (H&E) staining for evaluation of the morphology and regeneration of gastrocnemius muscle. The H&E staining images of muscle sections were recorded using Panoramic MIDI system (3DHISTECH Ltd.). Image‐Pro Plus software was used to analyse the mean muscle fibre cross‐sectional area (CSA).

### 2.7 Immunohistochemical Staining

After slides were dewaxed and hydrated, antigens were retrieved using a citric acid buffer in a microwave oven for 20 min. Endogenous peroxidases were inactivated by treating with 3% hydrogen peroxide for 10 min, and 5% bovine serum albumin was added to block non‐specific binding. Next, the tissue sections were incubated overnight at 4°C with primary antibodies. Subsequently, the sections were incubated with an enhancing solution at room temperature for 15 min and then with secondary antibodies at 37°C for 15 min. Sections were washed with phosphate‐buffered saline (PBS) for three times between each of the aforementioned steps. Lastly, 3,3′‐diaminobenzidine (DAB) solution (Wuhan Servicebio Technology G1212, China) was added, and the sections were examined under a microscope. When the target proteins turned yellow, the slides were transferred to distilled water, after which they were soaked in haematoxylin for 3 min, washed in running water for 30 s, subjected to differentiation in 1% hydrochloric acid ethanol for 1 s, dehydrated with ethanol, cleared in a dewaxing solution and mounted. After acquisition, images were analysed using Image‐Pro Plus.

### 2.8 Gene Expression Omnibus Analysis

We assessed the microarray data of limb muscles from six male *Mus musculus* in the Gene Expression Omnibus database (GSE132040). Three‐month‐old mice are the young group and 24‐month‐old mice are the aged group (*n* = 3).

### 2.9 Cell Culture

HEK293T cells were purchased from Procell Life Science& Technology Co. Ltd. The cells were cultured in DMEM with 10% foetal bovine serum and 1% penicillin/streptomycin (PWL062, Meilunbio, China) at 37°C with 5% CO_2_. Mouse skeletal muscle fibroblast cells (NOR‐10 cells) were presented by Prof. Xiao Qian of Chongqing Medical University. The cells were cultured in DMEM with 20% foetal bovine serum and 1% penicillin/streptomycin at 37°C with 5% CO_2_. Mouse primary skeletal muscle cells were purchased from ShareBio Science& Technology Co. Ltd. The cells were cultured in medium for mouse skeletal muscle cells (SB‐YMMP084) at 37°C with 5% CO_2_. For D‐gal–induced group, NOR‐10 cells and primary skeletal muscle cells were treated with different doses of D‐gal (D‐(+)‐gal, Sigma‐Aldrich, G5388) for 3 days at 50% confluence. Gentle handling was required when changing medium during D‐gal exposure. To test the effect of recombinant mouse decorin on D‐gal–induced senescent cells, different doses of decorin (Novoprotein, CM87) and the cells were co‐incubated with D‐gal for 72 h.

### 2.10 RNA Interference

The negative control (si‐NC) for in vitro RNA interference (RNAi) was obtained from GeneCreate Biological Engineering (Wuhan, China). All the specific siRNA sequences used for RNAi are listed in Table S1. NOR‐10 cells were seeded into 6‐ or 12‐well plates. After 12 h, the cells were transfected with siRNAs by using GP‐transfect‐Mate (Genepharma, Shanghai, China). After 6 h, the cells were washed twice with PBS and incubated in the DMEM medium containing 100 mM D‐gal for 3 days to induce NOR‐10 cell senescence.

### 2.11 Lentivirus Packaging

The coding sequence of the mouse Dcn gene was cloned into the lv‐plex expression vector to generate the Dcn expression vector (SYNBIO‐TECH). For lentivirus packing, HEK293T cells were co‐transfected with the lentiviral vectors psPAX2 (12 260, Addgene), PMD2.G (12 259, Addgene) and Dcn expression vector using lipo8000 Transfection Reagent (C0533, Beyotime). Viral particles were collected at 48 and 72 h after transfection. Then, the virus was concentrated using Universal Virus Concentration Kit (C2901S, Beyotime).

### 2.12 Lentivirus‐Mediated Expression of Dcn

NOR‐10 cells were incubated in medium containing optimal dilutions of lentivirus mixed with polybrene and changed with complete medium after 24 h of transfection. Then cells were subjected to puromycin selection (15 μg/mL) to obtain stably transfected cells for subsequent experiments.

### 2.13 Cell Viability

Cell viability was determined by cell counting kit‐8 assay (Meilunbio, MA0218). NOR‐10 cells were seeded in 96‐well plates at a density of 1 × 10^5^ cells per well. After being cultured in complete medium for 6 h, the fibroblasts were treated with different concentrations of D‐gal or decorin for 96 h. Then, cell viability was determined by incubation with DMEM containing CCK‐8 for 60 min. The absorbance at 450 nm was measured by microplate reader. Cell survival rate = [(OD of experimental wells − OD of blank wells) / (OD of control wells − OD of blank wells)] × 100%.

### 2.14 SA‐β‐Gal Staining

SA‐β‐gal staining was performed using a SA‐β‐gal staining kit (Beyotime Biotechnology, C0602). NOR‐10 cells were seeded in six‐well plates. SA‐β‐gal staining solution fixative was added to the cell medium, and the cells were fixed at room temperature for 15 min. Cells were then washed three times with PBS for 3 min each. Then, 1 mL SA‐β‐gal staining working solution was added to each well and incubated overnight at 37°C without CO_2_ until some of the cells turned blue. Cells were observed under the microscope and photographed, and positive cells were counted.

### 2.15 PCR and qRT‐PCR

Genomic DNA was extracted from toes using phenol (Solarbio, T0250) and trichloromethane was used for genotype identification. Total RNA was isolated from gastrocnemius tissues using TRNzol Universal (Tiangen, DP424) and reverse‐transcribed to single‐stranded cDNA using a reverse transcription system (Accurate Biology, AG11705). The qRT‐PCR was performed using 2 × SYBR Green Pro TaqHS Premix (Accurate Biology, AG11701) with an Applied Biosystems StepOnePlus Real‐Time PCR System, and PCR was performed on a TaKaRa PCR Thermal Cycler, according to the manufacturer's instructions. The primers used to detect the expression of target genes are shown in Table S2. A separate analysis using primers for the detection of RPL35A was used as an internal control.

### 2.16 Western Blotting

Western blotting procedure was performed according to literature report [28]. Briefly, gastrocnemius samples or treated fibroblast cells were lysed in ice‐cold RIPA buffer (Epizyme Biotech, PC105) with the PMSF protease inhibitor (Epizyme Biotech, GRF101) and phosphatase inhibitor cocktail (Epizyme Biotech, GRF102). The total protein concentration of the supernatant was determined using a BCA assay kit (Thermo Fisher Scientific, A55864). Equal amounts of proteins from different groups were resolved via 10% or 12% SDS–PAGE and then transferred onto NC membranes (Sangon Biotech, F619511–0005). Subsequently, the membranes were blocked for 2 h at RT with 5% milk in TBST and then incubated overnight at 4°C with primary antibodies (Table S3). Then, the membranes were washed three times with TBST and incubated with horseradish peroxidase–conjugated anti‐rabbit or anti‐mouse IgG antibodies at room temperature for 1 h. The target protein bands were visualised using an enhanced chemiluminescence high‐sensitivity reagent (MA0186, Meilunbio) and a ChemiDoc Imager (Micro Chemi). The densitometric values were determined using the ImageJ software (National Institutes of Health, USA). The relative protein levels were normalised against the levels of β‐actin or GAPDH, used as an internal control.

### 2.17 Immunofluorescence

The cover glasses were put in a 6‐well plate, and NOR‐10 cells(2.5 × 10^5^ cells) were seeded to grow overnight. After fixing with 4% paraformaldehyde for 15 min, the glasses were washed with PBS for three times, 5 min each. After being preincubated with goat serum for 60 min, the glasses were treated with the combination of anti–α‐SMA (#40482, SAB, 1:200) overnight at 4°C. Then, the goat anti‐rabbit IgG H&L (Abcam, ab150077, 1:500) and Cy3‐labelled goat anti‐mouse IgG H&L (A0521, Beyotime, 1:500) were incubated in the dark for 60 min. The glasses were washed again with washing solution for three times, 5 min each time. The anti‐fluorescence quenching sealing liquid was dropped on the glass slide, and the cover glass with cells was covered to avoid bubbles as far as possible. The glasses were observed, and images were obtained with an inverted fluorescence microscope (Olympus IX73, Japan).

### 2.18 Co‐Immunoprecipitation

Cells on ice were lysed using a buffer provided with co‐immunoprecipitation kits containing protease inhibitors (JKR23001A, Wuhan), as described by the manufacturer. Lysate containing 1 mg of total protein mixed with 3 μg of anti‐decorin (Abcam, ab175404) or anti‐ITGB1 (Santa Cruz, sc‐53 711) was then incubated with gentle shaking overnight at 4°C. Protein A/G Magnetic Beads were added to each tube, and the samples were incubated again with gentle shaking at RT. The immunocomplex was washed with cold elution buffer and the antibody‐selected proteins were eluted from the agarose beads by boiling in SDS‐loading buffer (0.1 M Tris–HCl, 10% glycerol, 2% SDS, 0.05% bromophenol blue and 0.1 M DTT) for 10 min. Each sample was resolved on a 10% SDS–PAGE and visualised using an enhanced chemiluminescence high‐sensitivity reagent. The positive control was input.

### 2.19 Puromycin Assay

SUNSET method was used to measure protein synthesis in vitro. For the in vitro study, 10 μg/mL puromycin was added to the medium 1 h before NOR‐10 was collected. The incorporation of puromycin in the total protein was analysed by Western blotting.

### 2.20 Statistical Analysis

All experiments were repeated at least three times, and data are presented as mean ± SD. The differences between the two groups were evaluated using Student's *t* test. Multiple group comparisons were performed using one‐way ANOVA. *p* < 0.05 was considered statistically significant. Statistical analyses were conducted using GraphPad Prism 9.0 (San Diego, CA, USA).

## 3 Results

### 3.1 Reduced Expression Levels of Decorin Were Found in Natural Ageing Animal Model

**FIGURE 1:**
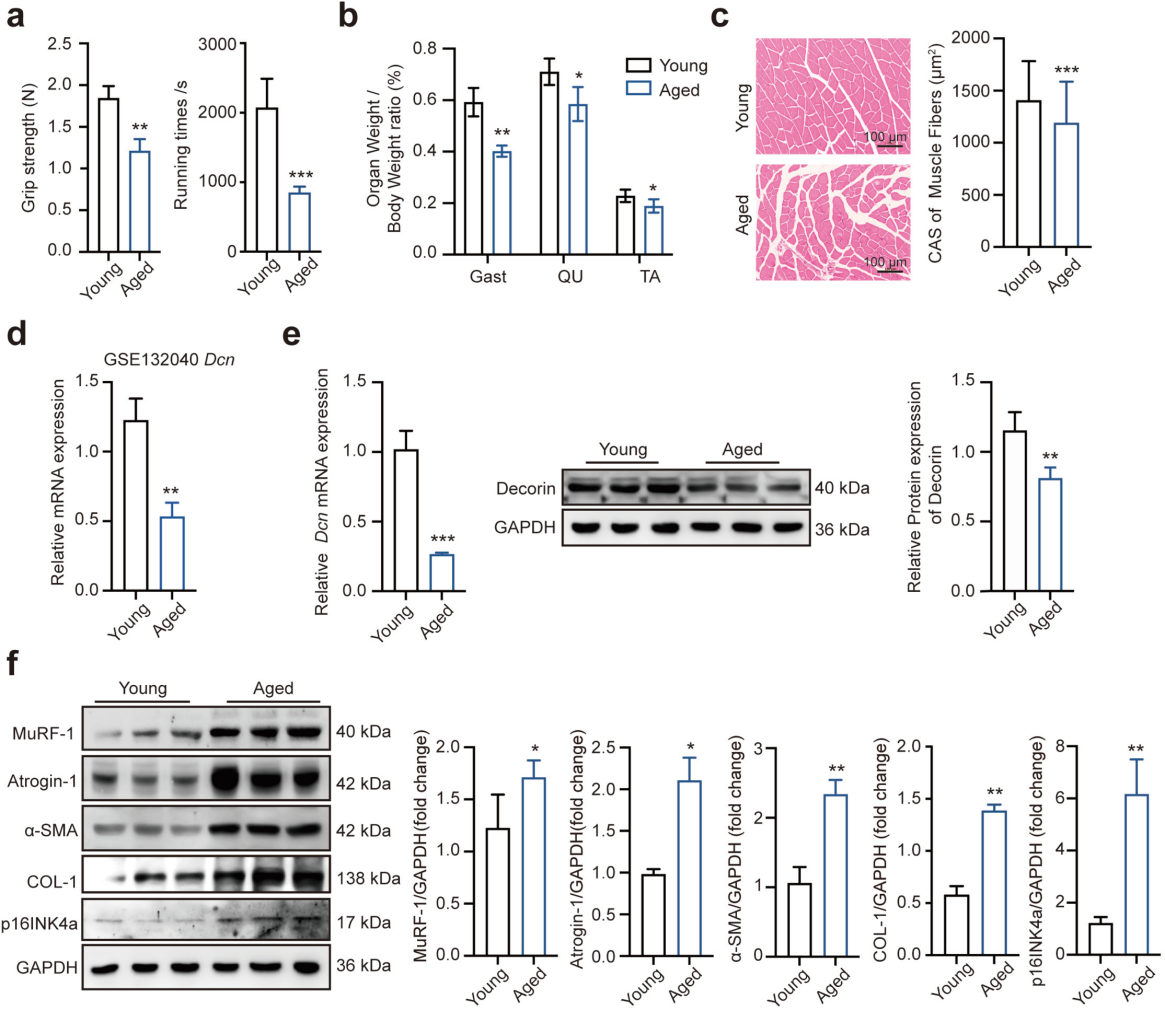
The expression levels of decorin were found reduced in muscles during ageing. (a) The forelimb grip strength and running times in young and aged group mice (*n* = 6). (b) The ratio of gastrocnemius (Gast), quadriceps femoris (QU) and tibialis anterior (TA) weights/body weight (*n* = 6). (c) Haematoxylin and eosin histological analysis and average cross‐sectional area of fibre sizes (*n* = 6). (d) Dcn expression levels in young and aged mice in GSE132040 (*n* = 3). (e) qRT‐PCR and Western blotting analysed the decorin protein and mRNA levels in gastrocnemius from 3‐ and 18‐month‐old mice (*n* = 6). (f) Western blotting analysed the expression of p16INK4a, MuRF‐1, Atrogin‐1, α‐SMA and COL‐1 proteins (*n* = 6). GAPDH: internal reference. Scale bar, 100 μm. Data are presented as mean ± SD, *p < 0.05, **p < 0.01, ***p < 0.001 compared with the young group. ns = no significant difference.

Natural ageing animal model was established by raising C57BL/6 J mice until 18 months old. Aged mice had significantly reduced grip strength and running times compared with young group mice (Figure 1a). The ratio of gastrocnemius (Gast), quadriceps (QU) and tibialis anterior (TA) weights/body weight was decreased compared with young group mice (Figure 1b). Meanwhile, muscle fibre areas were reduced in aged group mice (Figure 1c). Interestingly, *Dcn* mRNA expression was reduced in Limb_muscles of aged mice compared with young mice in GSE132040 (Figure 1d). Furthermore, we found that decorin expression markedly decreased in aged group mice (Figure 1e). The protein levels of age‐associated markers including p16INK4a, MuRF‐1 and Atrogin‐1, as well as α‐SMA and COL‐1 in gastrocnemius, were significantly higher in aged group mice (Figure 1f). These findings reveal that decorin might contribute to the physiological process of skeletal muscle ageing.

### 3.2 siRNA Knockdown Dcn Promotes Senescence and Fibrosis in D‐Gal–Induced NOR‐10 Cells

**FIGURE 2:**
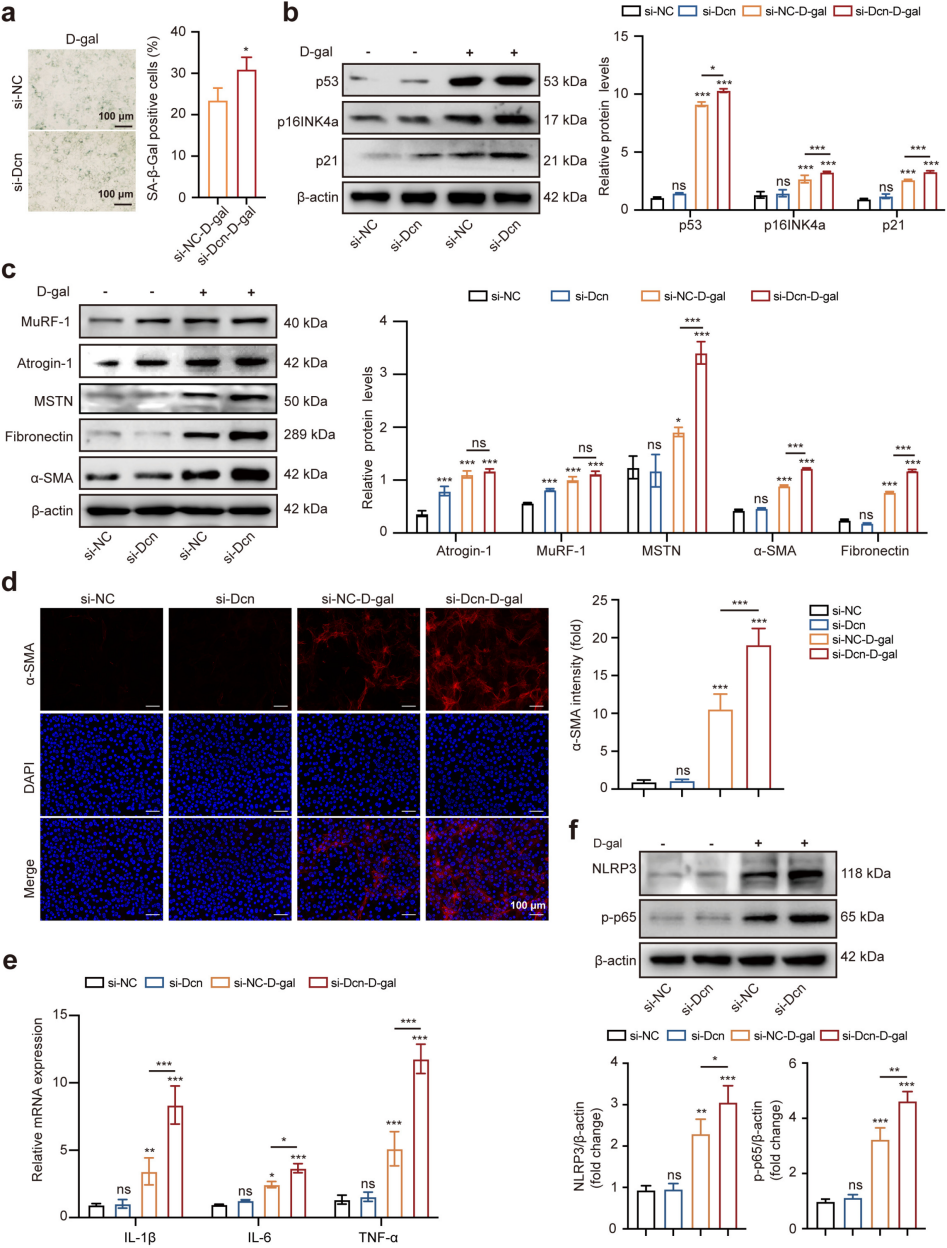
si‐Dcn exacerbated senescence and fibrosis in D‐gal–induced NOR‐10 cells. (a) SA‐β‐gal staining and quantification in D‐gal–induced NOR‐10 cells (*n* = 3). (b) Protein levels of p53, p16INK4a and p21 in si‐NC, si‐Dcn, si‐NC–D‐gal, si‐Dcn–D‐gal groups (*n* = 3). (c) Protein levels of MuRF‐1, Atrogin‐1, MSTN, fibronectin and α‐SMA in D‐gal–induced NOR‐10 cells of knockdown Dcn; the relative protein levels of the target proteins were normalised to those of β‐actin (*n* = 3). (d) Immunofluorescence staining of α‐SMA in si‐NC, si‐Dcn, si‐NC–D‐gal and si‐Dcn–D‐gal groups (*n* = 3). (e) Relative mRNA levels of IL‐6, IL‐1β and TNF‐α in D‐gal–induced NOR‐10 cells of knockdown Dcn. (f) Protein levels of NLRP3 and p‐p65 in D‐gal–induced NOR‐10 cells of knockdown Dcn. Scale bar, 100 μm. Data are presented as mean ± SD, and *p < 0.05, **p < 0.01, ***p < 0.001 compared with si‐NC group. ns = no significant difference.

NOR‐10 cells are a classical cell line of mouse skeletal muscle fibroblasts, and there is a clear literature to support their use in fibrosis studies [7, 29, 30, 31]. NOR‐10 cells treated with 100 mM D‐gal for 72 h were induced to senescence (Figure S1a,b). The expression of decorin was found significantly reduced in NOR‐10 cells after D‐gal–induced senescence (Figure S1c). To further elucidate the function of decorin in NOR‐10 cells' ageing, we knocked down *Dcn* in NOR‐10 cells and then induced cell ageing by using 100 mM D‐gal treatment for 72 h. The Dcn‐knockdown group (si‐Dcn) remarkably reduced the expression of decorin compared with the negative control group (si‐NC) (Figure S1d). SA‐β‐gal staining showed the number of SA‐β‐gal–positive cells gradually increased in the si‐Dcn–D‐gal group (Figure 2a), indicating decorin deficiency significantly accelerated the senescence of D‐gal–induced NOR‐10 cells. Further, the protein levels of p53, p21 and p16INK4a, the factors associated with senescence, were significantly increased in the si‐Dcn–D‐gal group compared with the si‐NC–D‐gal group as shown in Figure 2b. Meanwhile, the skeletal muscle atrophy proteins (MuRF‐1, Atrogin‐1, MSTN) and fibrosis proteins (fibronectin, α‐SMA) were also found dramatically increased in the si‐Dcn–D‐gal group (Figure 2c). As expected, the fluorescence intensity of α‐SMA was significantly increased in the si‐Dcn–D‐gal group (Figure 2d). These data indicated that decorin deficiency might exacerbate senescence and fibrosis in D‐gal–induced NOR‐10 cells. In addition, the mRNA expression levels of inflammation‐related genes (IL‐6, IL‐1β and TNF‐α) and proteins of NLRP3 and p‐p65 were significantly increased in the si‐Dcn–D‐gal group compared with the si‐NC–D‐gal group (Figure 2e,f), suggesting that Dcn knockdown could promote the occurrence of inflammation.

### 3.3 Decorin Deficiency Accelerates Skeletal Muscle Wasting and Fibrosis in D‐Gal–Induced Ageing Mice

**FIGURE 3:**
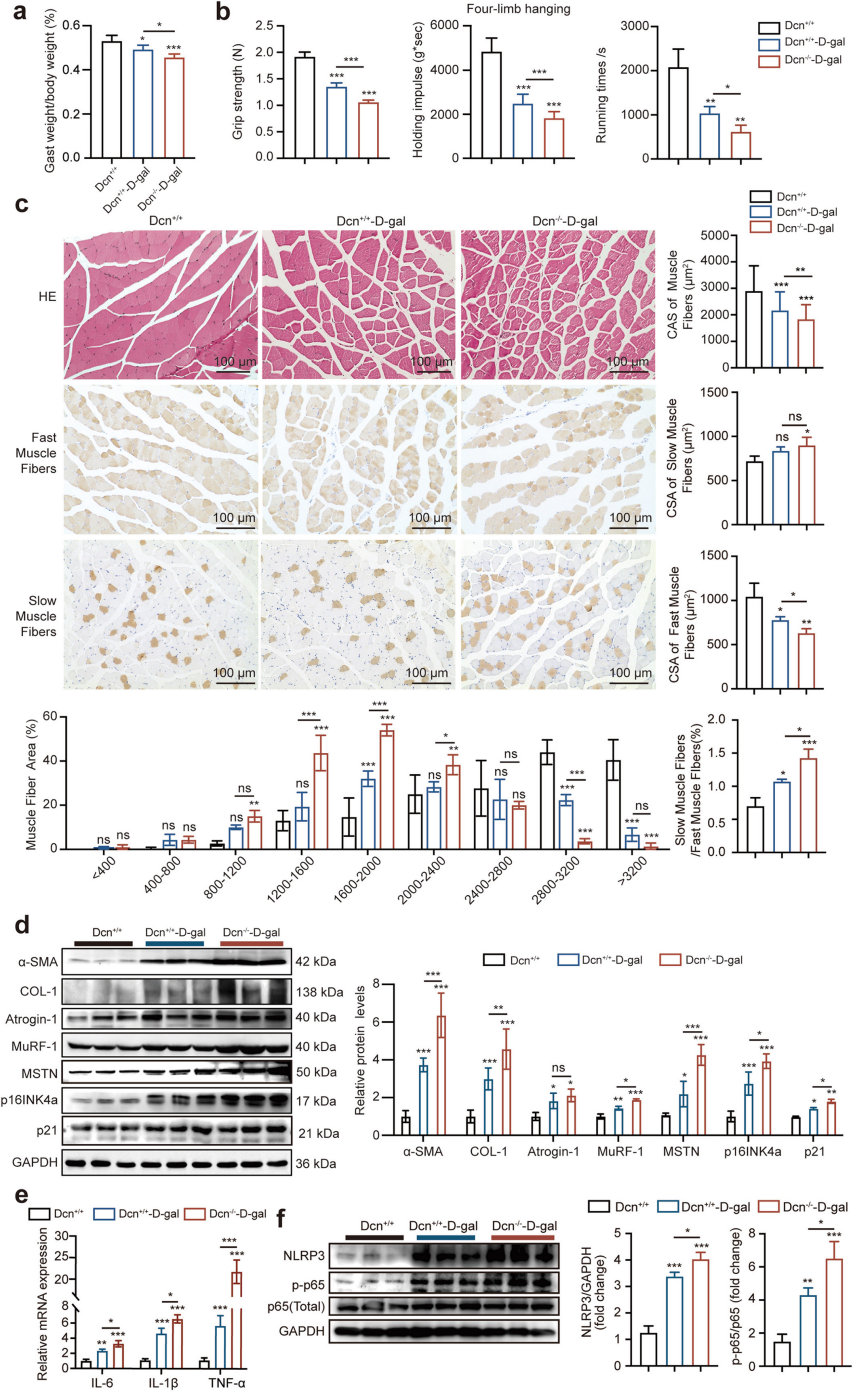
Decorin deficiency in D‐gal–induced aged mice exacerbated skeletal muscle wasting. (a) The ratio of Gast/body weight in Dcn^+/+^, Dcn^+/+^–D‐gal and Dcn^−/−^–D‐gal groups (*n* = 6). (b) The grip strength, muscular strength and running times of mice were analysed by electronic dynamometer, four‐limb hanging test and treadmill (*n* = 6). (c) Representative H&E, fast muscle fibres and slow muscle fibres staining, quantifications of muscle fibre sizes distribution, average cross‐sectional areas (CSAs) and CSA of slow/fast muscle fibres (*n* = 6). (d) Western blotting analysed the protein levels of α‐SMA, COL1, MuRF‐1, Atrogin‐1, MSTN, p16INK4a and p21 in Gast muscles (*n* = 3). (e) Relative mRNA levels of IL‐6, IL‐1β and TNF‐α genes in Gast muscles. (f) Western blotting analysed the protein levels of NLRP3, p‐p65 and p65 in Gast muscles. Scale bar, 100 μm. Data are presented as mean ± SD and *p < 0.05, **p < 0.01, ***p < 0.001 compared with Dcn^+/+^ group. ns = no significant difference.

To further investigate the function of decorin in muscle during ageing, a Dcn^+/+^ and Dcn^−/−^ ageing mouse model was established by using 500 mg/kg D‐gal, which significantly reduced the body weight compared with the saline group (Dcn^+/+^ group) after 10 weeks of treatment and which showed more prominent performance in the Dcn^−/−^–D‐gal group (Figure S2b,c). In addition, the muscle weight (Figure S2d) and the ratio of Gast/body weight were also greatly reduced in the Dcn^−/−^–D‐gal group (Figure 3a). The grip force, holding impulse and running times were significantly reduced in the Dcn^+/+^ or Dcn^−/−^–D‐gal groups compared with the Dcn^+/+^ group, and impressively, the decline of grip force and holding impulse was observed in the Dcn^−/−^–D‐gal group (Figure 3b). Consistently, H&E and immunohistochemical staining indicated that the CSA of muscle fibres and fast muscle fibres were significantly reduced in the Gast muscle of the Dcn^−/−^–D‐gal group, and the percentage of slow/fast muscle fibres was increased in the Dcn^−/−^–D‐gal group compared with the Dcn^+/+^ group (Figure 3c). Accordingly, the expression levels of atrophic genes (Atrogin‐1, MuRF‐1 and MSTN), fibrosis‐related genes (α‐SMA and FN1) and the age‐associated gene p16INK4a were increased in the Dcn^−/−^–D‐gal group (Figure S2e–g). Additionally, the expression levels of age‐associated proteins (p16INK4a, p21), atrophic‐associated proteins (MuRF‐1, Atrogin‐1, MSTN) and fibrosis‐associated proteins (COL1, α‐SMA) were evidently increased in Dcn^−/−^–D‐gal mice (Figure 3d). In addition, the expression levels of myogenic differentiation proteins were decreased in Dcn^−/−^–D‐gal mice (Figure S2h,i). Furthermore, the mRNA levels of IL‐6, IL‐1β, TNF‐α and protein levels of p‐p65 and NLRP3 were increased in Dcn^−/−^–D‐gal mice compared with Dcn^+/+^–D‐gal mice (Figure 3e,f). The data from Dcn^−/−^ mice suggested that decorin deficiency could cooperatively exacerbate skeletal muscle wasting in senescence.

### 3.4 Dcn Overexpression Reverses D‐Gal–Induced NOR‐10 Cell Senescence and Fibrosis

**FIGURE 4:**
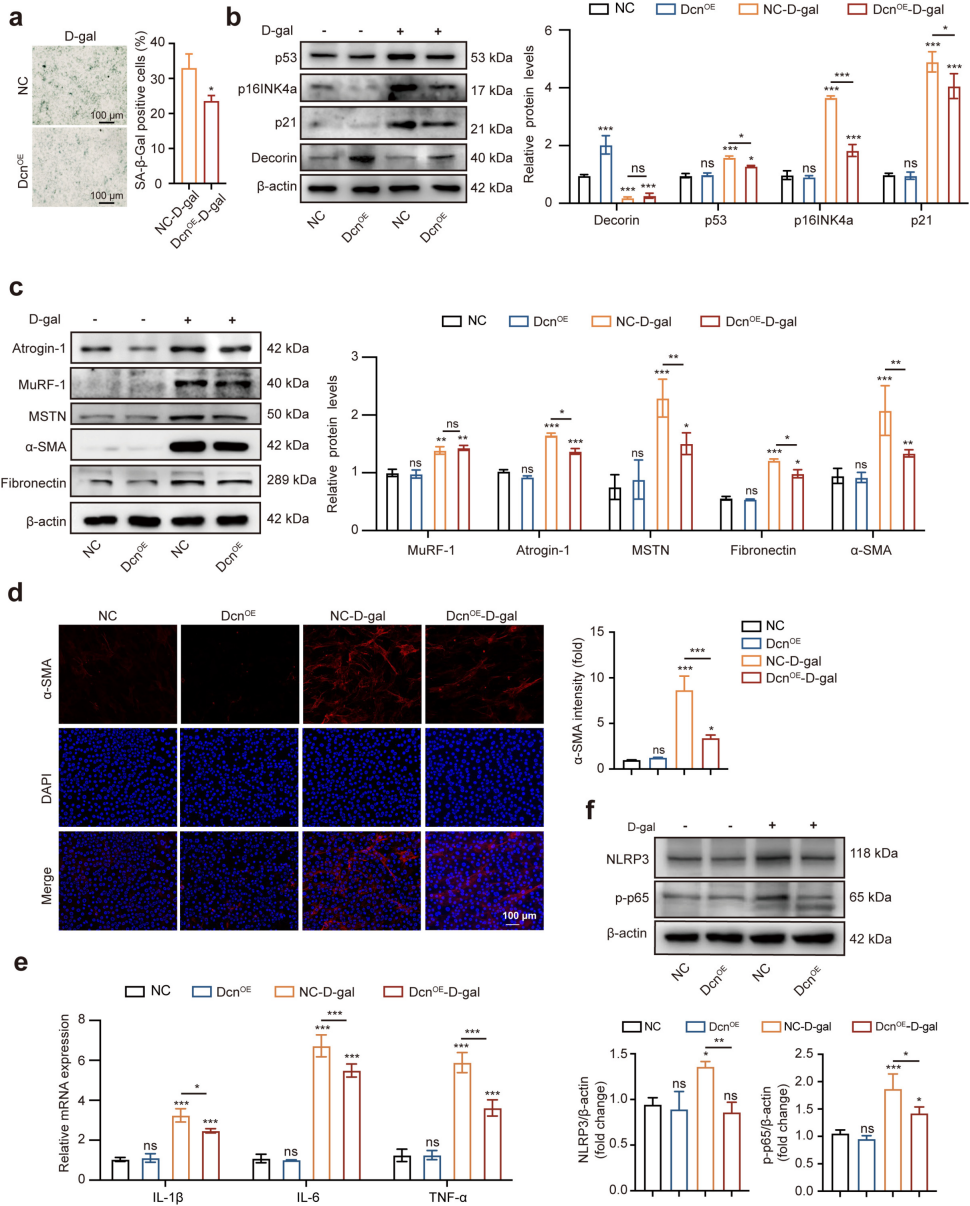
Dcn overexpression ameliorated age‐associated phenotypes of NOR‐10 cells induced by D‐gal. (a) SA‐β‐gal staining in NC–D‐gal and Dcn^OE^–D‐gal groups (*n* = 3). (b) Protein levels of p53, p16INK4a, p21 and decorin in NC, Dcn^OE^, NC–D‐gal and Dcn^OE^–D‐gal groups by Western blotting (*n* = 3). (c) Protein levels of Atrogin‐1, MuRF‐1, MSTN, fibronectin and α‐SMA in NC, Dcn^OE^, NC–D‐gal and Dcn^OE^–D‐gal groups by Western blotting; the relative protein levels of the target proteins were normalised to those of β‐actin (*n* = 3). (d) Immunofluorescence staining of α‐SMA in NC, Dcn^OE^, NC–D‐gal and Dcn^OE^–D‐gal groups (*n* = 3). (e) Relative mRNA levels of IL‐6, IL‐1β and TNF‐α genes in NC, Dcn^OE^, NC–D‐gal and Dcn^OE^–D‐gal groups. (f) Western blotting analysed the protein levels of NLRP3 and p‐p65 in NC, Dcn^OE^, NC–D‐gal and Dcn^OE^–D‐gal groups. Scale bar, 100 μm. Data are presented as mean ± SD and *p < 0.05, **p < 0.01, ***p < 0.001 compared with NC and Dcn^OE^ group. ns = no significant difference.

Considering the important function of decorin in ageing, we constructed Dcn‐overexpressing NOR‐10 cells with lentivirus to investigate whether decorin could protect NOR‐10 cells against D‐gal–induced senescence and fibrosis. qRT‐PCR and Western blotting demonstrated a significant increase in decorin expression in the Dcn‐overexpressing (Dcn^OE^) group compared with the negative control lentivirus (NC) groups (Figure S3a,b). As anticipated, SA‐β‐gal staining showed the number of SA‐β‐gal–positive cells was reduced in the Dcn^OE^–D‐gal group (Figure 4a). In addition, the results showed that Dcn overexpression reduced the levels of p53, p21 and p16INK4a proteins induced by D‐gal (Figure 4b). Moreover, the protein levels of skeletal muscle atrophy–associated proteins (MuRF‐1, Atrogin‐1, MSTN) and fibrosis‐related proteins (fibronectin, α‐SMA) were decreased in the Dcn^OE^–D‐gal group compared with the NC–D‐gal group (Figure 4c). As expected, the fluorescence intensity of α‐SMA was significantly decreased in the Dcn^OE^–D‐gal group (Figure 4d). Collectively, our findings suggested that Dcn‐overexpressing could protect NOR‐10 cells against D‐gal–induced senescence and fibrosis. In addition, we investigated the effects of Dcn overexpression on NF‐κB pathway–related inflammation in D‐gal–induced NOR‐10 cells. Compared with the NC group, the mRNA levels of IL‐6, IL‐1β and TNF‐α, as well as the protein levels of p‐p65 and NLRP3, were elevated in the NC–D‐gal group, while the expression levels of these proteins were significantly reduced in the Dcn^OE^–D‐gal group (Figure 4e,f), suggesting that Dcn overexpression could extenuate the inflammation.

### 3.5 Recombinant Mouse Decorin Alleviates D‐Gal–Induced NOR‐10 Cell Senescence and Fibrosis

**FIGURE 5:**
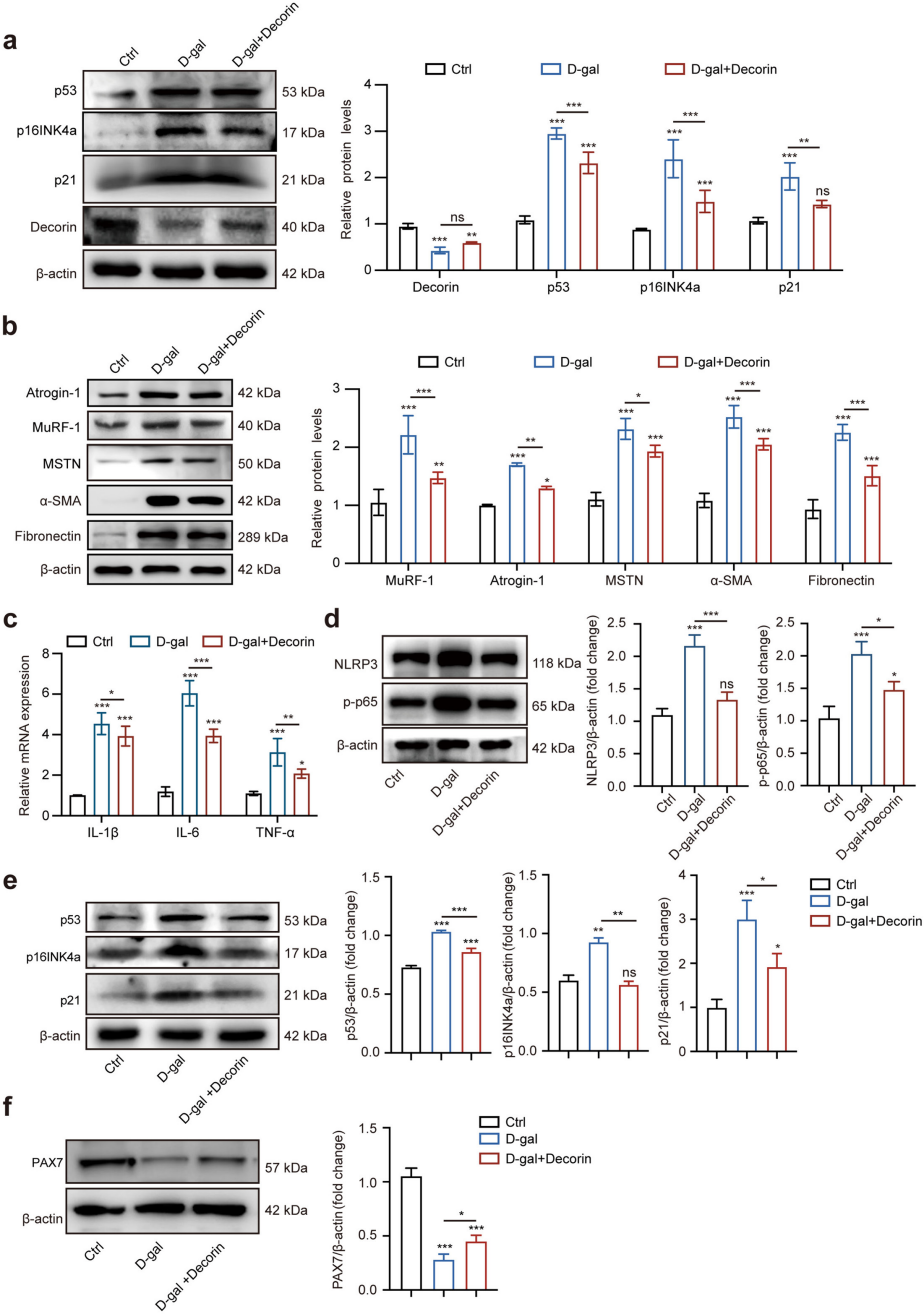
Recombinant decorin attenuated senescence and fibrosis in D‐gal–induced NOR‐10 cells and primary skeletal muscle cells. (a) Western blotting analysed the protein levels of p16INK4a, p53, p21 and decorin in Ctrl, D‐gal and D‐gal + decorin groups (*n* = 3). (b) Western blotting analysed the protein levels of Atrogin‐1, MuRF‐1, MSTN, fibronectin and α‐SMA in Ctrl, D‐gal and D‐gal + decorin groups (*n* = 3). (c) Relative mRNA levels of IL‐6, IL‐1β and TNF‐α genes in Ctrl, D‐gal and D‐gal + decorin groups. (d) Western blotting analysed the protein levels of NLRP3 and p‐p65 in Ctrl, D‐gal and D‐gal + decorin groups. (e) Western blotting analysed the protein levels of p53, p16INK4a and p21 in Ctrl, D‐gal and D‐gal + decorin groups. (f) Western blotting analysed the protein levels of PAX7 in Ctrl, D‐gal and D‐gal + decorin groups. Scale bar, 100 μm. Data are presented as mean ± SD and *p < 0.05, **p < 0.01, ***p < 0.001 compared with Ctrl group. ns = no significant difference.

To further determine the possible effect of recombinant decorin on D‐gal–induced NOR‐10 cells, D‐gal–induced NOR‐10 cells were incubated with different concentrations of recombinant decorin for 72 h, and then cell survival was tested, and 10 ng/mL recombinant decorin was chosen as the experimental concentration (Figure S4a,b). SA‐β‐gal staining showed the number of SA‐β‐gal–positive cells was significantly reduced in the D‐gal + decorin group compared with the D‐gal group (Figure S4c), which indicates that decorin significantly improved the senescence of D‐gal–induced NOR‐10 cells. Consistently, D‐gal upregulated the expression of fibrosis, atrophy and ageing genes and proteins, and recombinant decorin treatment significantly downregulated these markers (Figure 5a,b and S4d). Meanwhile, the fluorescence intensity of α‐SMA was significantly decreased in the D‐gal + decorin group (Figure S4e,f), suggesting that recombinant decorin could attenuate the atrophy and fibrosis in D‐gal–induced NOR‐10 cells. In addition, the mRNA levels of IL‐6, IL‐1β, TNF‐α and protein levels of p‐p65 and NLRP3 were reduced in the D‐gal + decorin group compared with the D‐gal group (Figure 5c,d). At the same time, we used primary skeletal muscle cells to detect the effect of recombinant decorin on D‐gal–induced ageing. Consistently, the mRNA and protein levels of p53, p21 and p16INK4a were decreased in the D‐gal + decorin group compared with the D‐gal group (Figure 5e and S4j,k), and the PAX7 protein was significantly increased in the D‐gal + decorin group compared with the D‐gal group (Figure 5f), indicating that recombinant decorin could alleviate ageing and activate satellite cells.

### 3.6 Akt/mTOR Signalling Pathway Was Involved in Regulation of Decorin on Skeletal Muscle Fibrosis and Atrophy

**FIGURE 6:**
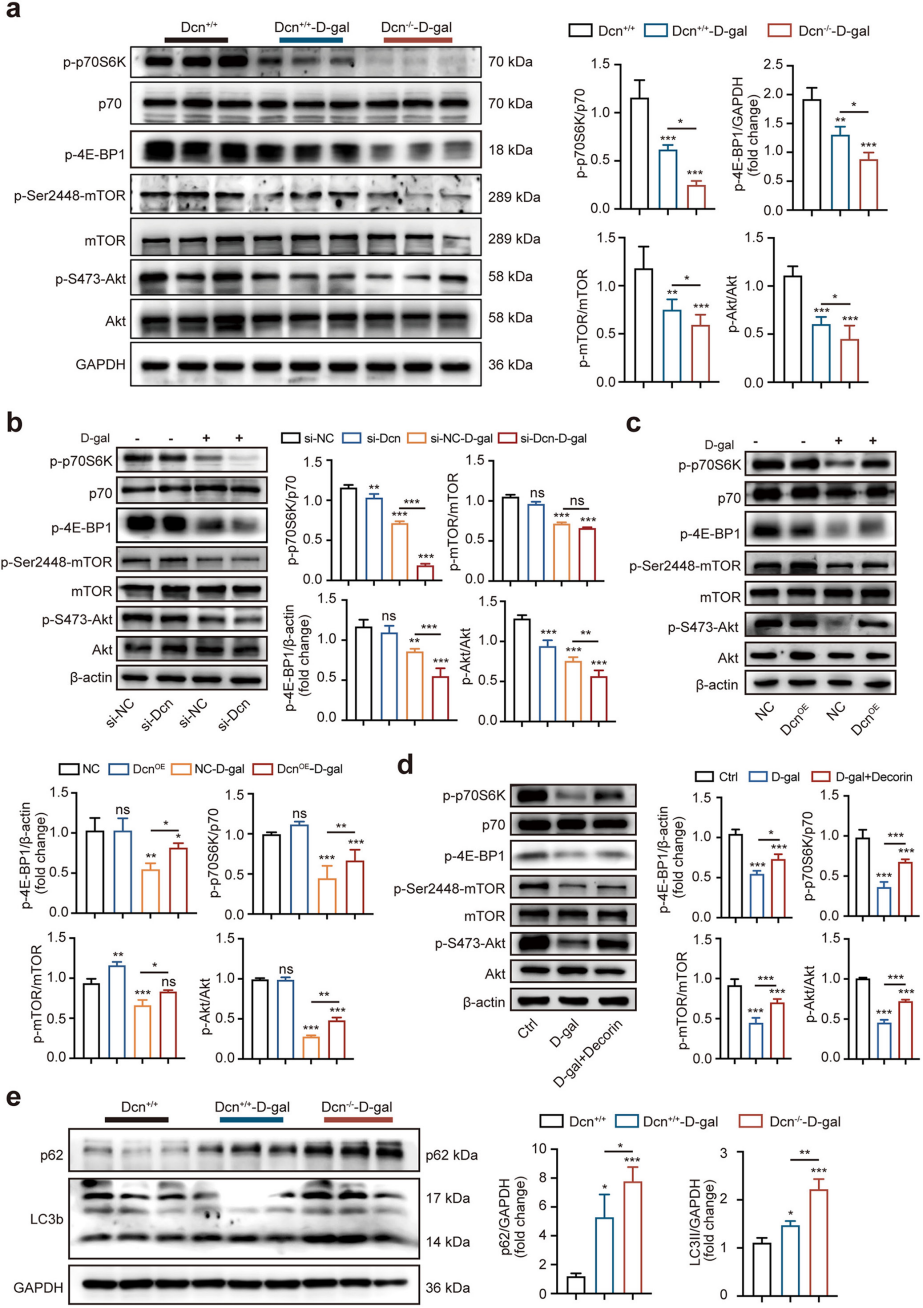
Akt/mTOR signalling pathway was involved in the regulation of decorin on skeletal muscle fibrosis and atrophy. (a) Western blotting analysed the expression levels of Akt, p‐S473‐Akt, mTOR, p‐Ser2448‐mTOR, p70, p‐p70S6K, p‐4E‐BP1, GAPDH and the corresponding statistical analysis in the Dcn^+/+^, Dcn^+/+^–D‐gal or Dcn^−/−^–D‐gal groups (*n* = 3). (b) Western blotting analysed the expression protein levels of Akt, p‐S473‐Akt, mTOR, p‐Ser2448‐mTOR, p70, p‐p70S6K, p‐4E‐BP1 and β‐actin and the corresponding statistical analysis in the si‐NC, si‐Dcn, si‐NC–D‐gal and si‐Dcn–D‐gal groups (*n* = 3). (c) Western blotting analysed the expression levels of Akt, p‐S473‐Akt, mTOR, p‐Ser2448‐mTOR, p70, p‐p70S6K, p‐4E‐BP1 and β‐actin and the corresponding statistical analysis in the NC, Dcn^OE^, NC–D‐gal and Dcn^OE^–D‐gal groups (*n* = 3). (d) Western blotting analysed the expression levels of Akt, p‐S473‐Akt, mTOR, p‐Ser2448‐mTOR, p70, p‐p70S6K, p‐4E‐BP1 and β‐actin and the corresponding statistical analysis in the Ctrl, D‐gal and D‐gal + decorin groups (*n* = 3). (e) Western blotting analysed the expression levels of p62, LC3b, GAPDH and the corresponding statistical analysis in the Dcn^+/+^, Dcn^+/+^–D‐gal or Dcn^−/−^–D‐gal groups (*n* = 3). Data are presented as mean ± SD and *p < 0.05, **p < 0.01, ***p < 0.001 compared with Dcn^+/+^, si‐NC, NC and Ctrl groups, respectively. ns = no significant difference.

PI3K/Akt/mTOR signalling pathway is a potential therapeutic target for reducing fibrosis development in sarcopenia [29]. Next, we investigated whether decorin regulates skeletal muscle fibrosis and atrophy associated with the PI3K/Akt/mTOR signalling pathway or not. Western blotting results showed that the p‐S473‐Akt, p‐Ser2448‐mTOR, p‐p70S6K and p‐4E‐BP1 proteins were markedly decreased in the Dcn^−/−^–D‐gal group (Figure 6a). Meanwhile, we found p‐S473‐Akt/Akt, p‐Ser2448‐mTOR/mTOR, p‐p70S6K/p70 and p‐4E‐BP1 were significantly reduced in the si‐Dcn–D‐gal group (Figure 6b). It revealed that decorin deficiency could inhibit the activation of the Akt/mTOR signalling pathway. Conversely, as shown in Figure 6c,d, compared with the NC or Ctrl group, Dcn overexpression or recombinant decorin partly returned the expression of p‐S473‐Akt, p‐Ser2448‐mTOR, p‐p70S6K and p‐4E‐BP1 proteins. Moreover, the expression of puromycin protein was increased in the Dcn^OE^–D‐gal group compared with the NC–D‐gal group (Figure S3c,d). Numerous studies have shown that mTORC1 is a key regulator of autophagy. The autophagy receptor p62 binds with LC3‐autophagosomes and promotes the degradation of abnormal proteins (LC3, p62), and the content of p62 is negatively correlated with the level of autophagy. Western blotting results showed that the levels of LC3‐II and p62 proteins were significantly higher in the Dcn^−/−^–D‐gal group than in the Dcn^+/+^ group (Figure 6e). In addition, the protein expression levels of LC3‐II and p62 were elevated in the si‐dcn group compared with si‐NC (Figure S1e,f). Conversely, overexpression of Dcn or recombinant decorin could reverse the protein expression levels of LC3‐II and p62 (Figure S3e,f and S4g–i). Thus, these data showed that decorin could act on aged mice or NOR‐10 cells to activate the Akt/mTOR signalling pathway and enhance protein synthesis.

### 3.7 Decorin Regulated Fibrosis and Atrophy in D‐Gal–Induced Senescence via ITGB1

**FIGURE 7:**
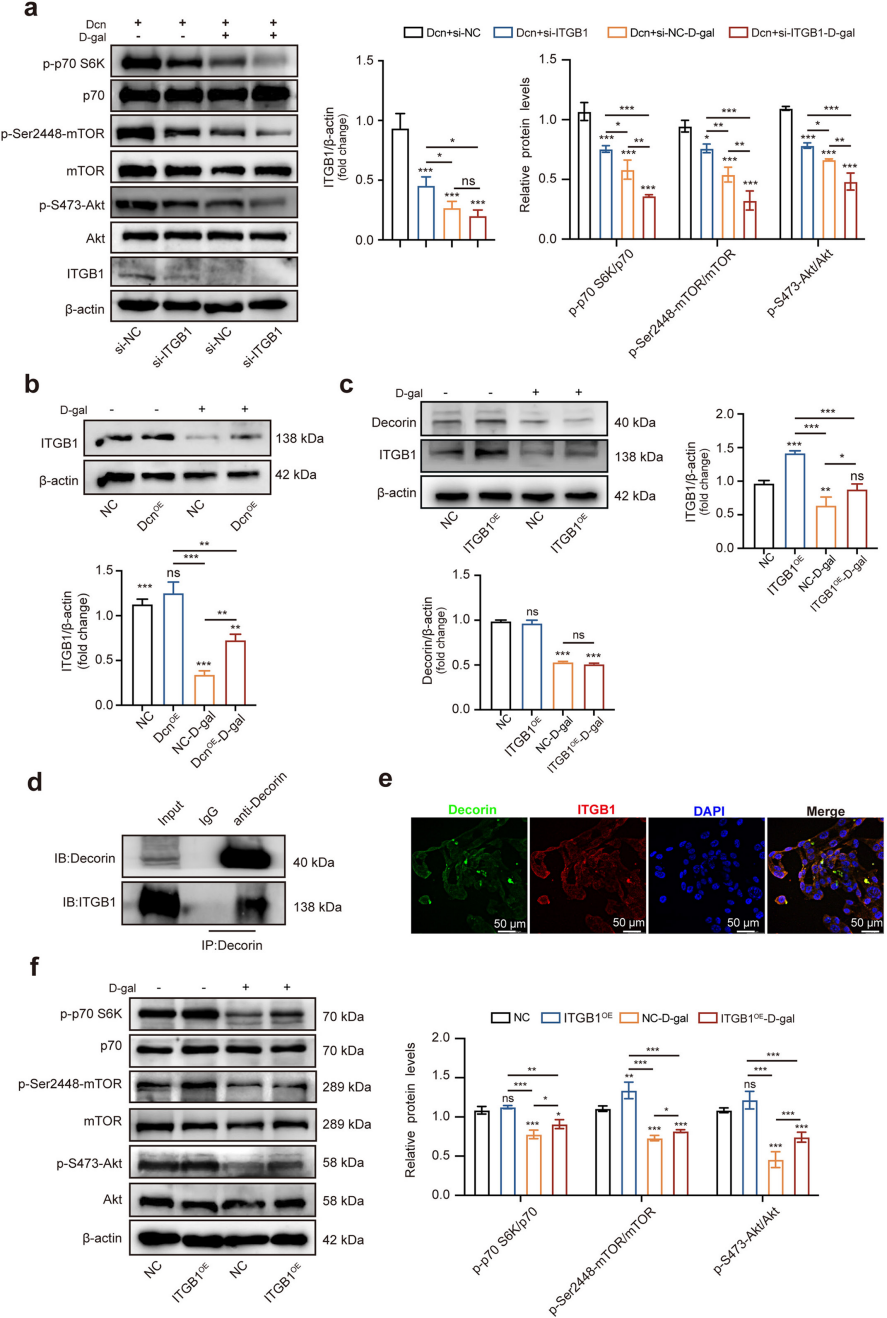
ITGB1 was involved in the regulation of fibrosis and atrophy by decorin. (a) Western blotting analysed the expression levels of Akt, p‐S473‐Akt, mTOR, p‐Ser2448‐mTOR, p70, p‐p70S6K and β‐actin and the corresponding statistical analysis in Dcn + si‐NC, Dcn + si‐ITGB1, Dcn + si‐NC–D‐gal and Dcn + si‐ITGB1–D‐gal groups (*n* = 3). (b) Western blotting analysed the effect of ITGB1 expression in NC, Dcn^OE^, NC–D‐gal and Dcn^OE^–D‐gal groups (*n* = 3). (c) Western blotting analysed the effect of ITGB1 expression in NC, ITGB1^OE^, NC–D‐gal and ITGB1^OE^–D‐gal groups (*n* = 3). (d) Lysates of NOR‐10 cells were immunoprecipitated with decorin antibody, and then Western blotting was performed with anti‐ITGB1 (*n* = 3). (e) Representative images of decorin and ITGB1 expression in NOR‐10 cells obtained by co‐immunofluorescence staining (*n* = 3). (f) Western blotting analysed the expression levels of Akt, p‐S473‐Akt, mTOR, p‐Ser2448‐mTOR, p70, p‐p70S6K and β‐actin and the corresponding statistical analysis in the NC, ITGB1^OE^, NC–D‐gal and ITGB1^OE^–D‐gal groups (*n* = 3). Scale bar, 50 μm. Data are presented as mean ± SD and *p < 0.05, **p < 0.01, ***p < 0.001 compared with si‐NC or NC group. ns = no significant difference.

ITGB1 activates intracellular signalling pathways that it mediates, such as the FAK pathway, the MAPK pathway and the PI3K‐Akt pathway [32]. To investigate whether decorin affects ITGB1 expression, the expression of ITGB1 in mice was examined by Western blotting. We found that ITGB1 expression was decreased in the Dcn^−/−^–D‐gal–induced group (Figure S5a). In our study, we knocked down ITGB1 in NOR‐10 cells with overexpression of Dcn (Figure S6a,b). si‐ITGB1 compromised decorin's regulation on Akt/mTOR signalling pathway–associated factors (Figure 7a). To verify the correlation between decorin and ITGB1, Dcn‐overexpressing NOR‐10 cells were induced by D‐gal to senescence. The expression of ITGB1 protein was increased in the Dcn^OE^–D‐gal group compared with the NC–D‐gal group (Figure 7b). Conversely, ITGB1 overexpression did not alter decorin expression (Figure 7c), which indicated that decorin acts as an upstream regulator for ITGB1. Then, we immunoprecipitated decorin in NOR‐10 cells and found that ITGB1 was present in the precipitate (Figure 7d). Also, co‐staining of decorin and ITGB1 revealed their co‐localisation in NOR‐10 cells (Figure 7e), suggesting there is a direct interaction between decorin and ITGB1. Besides, the expression of p‐S473‐Akt/Akt, p‐Ser2448‐mTOR/mTOR and p‐p70S6K/p70 in response to D‐gal could be reversed after ITGB1 overexpression (Figure 7f). In conclusion, these findings showed that decorin regulated fibrosis and atrophy in D‐gal–induced NOR‐10 cells senescence via ITGB1.

## 4 Discussion

Sarcopenia is a prominent phenomenon associated with ageing that the main mechanism represents loss of proteostasis, mitochondrial dysfunction and inflammatory disturbances [33]. In the present study, we found that Dcn knockout mice exacerbated muscle atrophy and decreased the grip force and CAS areas in aged mice. Of note, decorin alleviated skeletal muscle fibrosis and atrophy in D‐gal–induced aged mice and NOR‐10 cells by activating the ITGB1/Akt/mTOR signalling pathway, which suggests that decorin supplementation may be a potent therapeutic strategy to combat age‐associated sarcopenia.

Accumulating evidence has revealed that myokines play a prominent role in age‐associated sarcopenia [31, 34]. The myokines can be released after muscle contraction or strength training and play important roles in the control of muscle mass, autocrine regulation of muscle metabolism and paracrine/autocrine regulation of other tissues and organs [35, 36, 37]. Decorin was reported as a myokine previously; similarly, we found that decorin deficiency could promote skeletal muscle fibrosis and atrophy by D‐gal–induced mice and NOR‐10 cell models, such as fibronectin, α‐SMA, MuRF‐1 and Atrogin‐1. Meanwhile, we found MSTN was reduced significantly in the Dcn^OE^–D‐gal and D‐gal + decorin groups. This would provide direct evidence regarding muscle mass regulation in the context of decorin. D‐gal, as an important compound for investigating age‐associated diseases, has been applied in age‐associated skeletal muscle diseases [38, 39, 40]. Our findings showed that the optimal concentration for inducing cellular ageing was 100 mM of D‐gal, and the dose for animal senescence is 500 mg/kg/d. We also noted a simultaneous decrease in decorin expression and an increase in muscle atrophy in D‐gal–induced ageing skeletal muscle and NOR‐10 cells. At the same time, decorin deficiency promoted the D‐gal–induced reduction in the CSA of skeletal muscle fibres in mice and promoted gastrocnemius muscle atrophy and fibrosis, suggesting that decorin may affect the function of satellite cells and FAPs directly or indirectly.

Decorin is known to regulate cellular behaviour by binding to cell‐surface receptors such as EFGR, CD44 and ITGB1 [41, 42]. Our study revealed that decorin promotes skeletal muscle protein synthesis and enhances muscle function by specifically activating ITGB1. Other ECM components, like laminins and SPARC‐related modular calcium‐binding protein 2 (Smoc2), also influence muscle regeneration through ITGB1, suggesting a pivotal role for ITGB1 in connecting ECM signalling to muscle protein synthesis [14, 15]. We found that decorin‐triggered ITGB1 activation subsequently initiated the Akt/mTOR pathways, thereby promoting skeletal muscle proliferation and muscle repair. It is worth noting that the Akt/mTOR pathways were established for their significance in skeletal muscle atrophy [38, 43, 44]. Our findings highlighted the crucial role of ITGB1 in decorin orchestrating the activation of the Akt/mTOR pathways during skeletal muscle protein synthesis. Moreover, the interaction of decorin with ITGB1 was found in our study, which provided a clue for understanding the regulatory mechanisms of decorin expression in the skeletal muscle of ageing mice. Interestingly, we noticed that AMPK is a key energy sensor involved in mitochondrial homeostasis and ageing, and the assessment of its activation status can provide insights into the protective effects of decorin on D‐gal–induced cellular energy metabolism.

Autophagy is a highly conserved programmed cell death process in the evolution of life, which is closely regulated by a series of autophagy‐related genes [45]. In chronic inflammatory states, autophagy may be activated to assist skeletal muscle cells in clearing damaged macromolecules and dysfunctional organelles due to inflammation. We found that the proteins of autophagy (LC3b, p62) and inflammation (p‐p65, NLRP3) were increased significantly in the si‐Dcn‐gal and Dcn^−/−^–D‐gal groups. In contrast, overexpression of Dcn or recombinant decorin significantly reduced D‐gal–induced LC3b, p62, p‐p65 and NLRP3 protein expression, indicating a marked decline in autophagy function and promotion of inflammation. Decorin deficiency substantially increased p62 and LC3 levels and inhibited autophagy function, which probably accounted for the promotion of skeletal muscle fibre atrophy in aged knockout mice.

**FIGURE 8:**
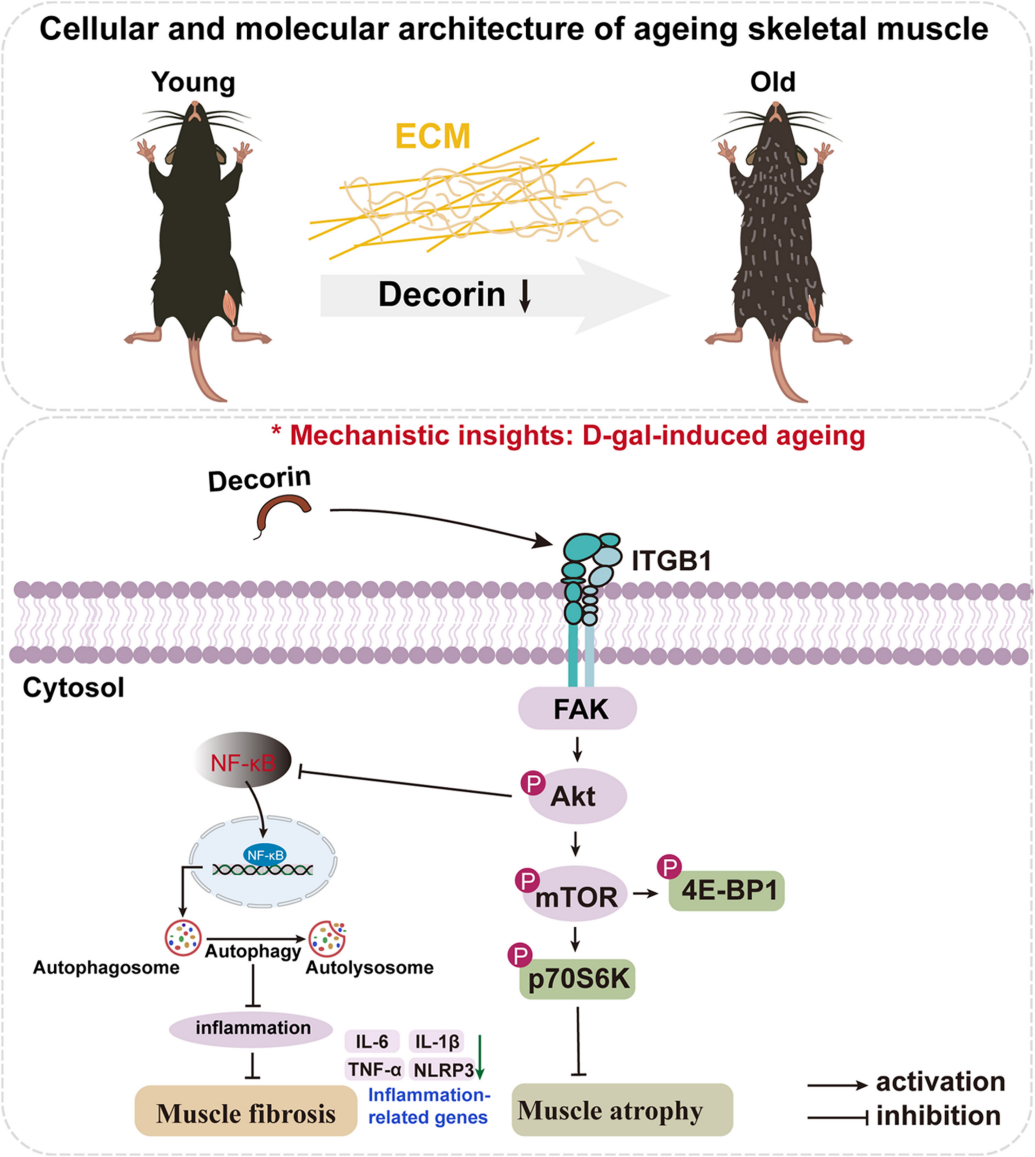
Schematic presentation of the decorin/ITGB1/Akt pathway.

Recombinant decorin protein was not applied in aged mice to evaluate its treatment efficiency on muscle atrophy and fibrosis; this is the limitation of the current study. In future studies, we will inject recombinant decorin into aged wild‐type mice and Dcn^−/−^ mice to verify the therapeutic effect of decorin on age‐related skeletal muscle fibrosis and atrophy. In addition, this study did not provide in vitro muscle contraction function data (such as tetanic and specific force) and precise morphological measurement data at the single muscle fibre level (such as single fibre CSA), which is the focus of our future study. Still, we believe that our study provides new insights into skeletal muscle ageing. The current study highlights that the activity of decorin‐mediated intracellular signalling was increased through the interaction between decorin and ITGB1, which is essential for decorin function during age‐associated muscle synthesis, as illustrated in Figure 8. These findings indicate that decorin could be a potentially valuable reagent for preventing and treating age‐associated sarcopenia.

## Funding

The authors received no specific funding for this work.

## Conflicts of Interest

The authors declare no conflicts of interest.

## Supporting information


**Table S1:** The sequences of siRNAs used for RNA interference and control.
**Table S2:** Sequences of primers used for PCR or qRT‐PCR amplification.
**Table S3:** Primary antibodies were utilised for Western blotting, immunofluorescence and co‐immunoprecipitation.
**Figure S1:** si‐Dcn exacerbated autophagy in D‐gal–induced NOR‐10 cells. (a) Effect of D‐gal (50–250 mM) on the proliferation of NOR‐10 cells. (b) Effect of different doses of D‐gal treatment on NOR‐10 cells viability at different periods. (c) Western blotting analysed the protein levels of decorin and β‐actin in D‐gal–induced NOR‐10 cells (*n* = 3). (d) The mRNA and protein levels of Decorin after knockdown Dcn in NOR‐10 cells (*n* = 3). (e) Protein levels of LC3b and p62 in D‐gal–induced NOR‐10 cells of knockdown Dcn. (f) The relative protein levels of the target proteins were normalised to those of β‐actin. All data are expressed as mean ± SD (*n* = 6) and *p < 0.05, **p < 0.01, ***p < 0.001 compared with the Ctrl and si‐NC groups.
**Figure S2:** Decorin deficiency exacerbated skeletal muscle wasting in D‐gal–induced mice. (a) The genotype of Dcn was identified by PCR analysis. (b) The changes body weight of Dcn^+/+^ mice with saline and Dcn^+/+^ and Dcn^−/−^ mice with D‐gal–induced groups (*n* = 6). (c) The body weight of Dcn^+/+^, Dcn^+/+^–D‐gal and Dcn^−/−^–D‐gal groups in 10 weeks. (d) The weights of quadriceps femoris (Qu), gastrocnemius (Gast) and tibialis anterior (TA) in Dcn^+/+^, Dcn^+/+^–D‐gal or Dcn^−/−^–D‐gal groups. (e) Relative mRNA levels of atrophic genes in Gast muscles. (f) Relative mRNA levels of age gene in Gast muscles. (g) Relative mRNA levels of fibrosis genes in Gast muscles. (h) Western blotting assays the protein levels of myogenin and MyoD1 in Gast muscles. (i) The relative protein levels of the target proteins were normalised to those of GAPDH. All data are expressed as mean ± SD (*n* = 6) and *p < 0.05, **p < 0.01, ***p < 0.001 compared with Dcn^+/+^ group.
**Figure S3:** Dcn overexpression reversed D‐gal–induced NOR‐10 cells autophagy. (a) Quantification of overexpression efficiency of Dcn in NOR‐10 cells by qRT‐PCR (*n* = 3). (b) Protein levels of decorin in NC and Dcn^OE^ groups by Western blotting (*n* = 3). (c) Western blotting analysed the protein levels of puromycin in NC, Dcn^OE^, NC–D‐gal and Dcn^OE^–D‐gal groups. (d) The relative protein levels of the target proteins were normalised to those of β‐actin. (e) Western blotting analysed the protein levels of p62 and LC3b in NC, Dcn^OE^, NC–D‐gal and Dcn^OE^–D‐gal groups. (f) The relative protein levels of the target proteins were normalised to those of β‐actin. All data are expressed as mean ± SD (*n* = 6) and *p < 0.05, **p < 0.01, ***p < 0.001 compared with NC group.
**Figure S4:** Recombinant decorin reduced autophagy in D‐gal–induced NOR‐10 cells. (a) Effect of 100 mM D‐gal and decorin (5–80 ng/mL) on the proliferation of NOR‐10 cells. (b) Effect of different doses of decorin treatment on NOR‐10 cell viability at different periods. (c) SA‐β‐gal staining and quantification in Ctrl, D‐gal and D‐gal + decorin groups (*n* = 3). (d) The mRNA levels of atrophic, fibrosis and ageing genes in Ctrl, D‐gal and D‐gal + decorin groups were measured by qRT‐PCR analysis (*n* = 3). (e) Immunofluorescence staining of α‐SMA in Ctrl, D‐gal and D‐gal + decorin groups. (f) The corresponding statistical analysis. (g) Western blotting analysed the protein levels of p62 and LC3b in Ctrl, D‐gal and D‐gal + decorin groups. (h, i) The relative protein levels of the target proteins were normalised to those of β‐actin. (j, k) Relative mRNA levels of ageing genes of primary skeletal muscle cells in Ctrl, D‐gal and D‐gal + decorin groups. Scale bar, 100 μm. All data are expressed as mean ± SD (*n* = 3) and *p < 0.05, **p < 0.01, ***p < 0.001 compared with Ctrl group.
**Figure S5:** Decorin deficiency reduced the expression of ITGB1 in D‐gal–induced aged mice. (a) Western blotting assays the protein levels of ITGB1, GAPDH and the corresponding statistical analysis in Dcn^+/+^, Dcn^+/+^–D‐gal or Dcn^−/−^–D‐gal groups. All data are expressed as mean ± SD (*n* = 3) and *p < 0.05, **p < 0.01, ***p < 0.001 compared with Dcn^+/+^ group.
**Figure S6:** The knockdown efficiency of siRNAs against ITGB1 in NOR‐10 cells with overexpression of Dcn. (a, b) The mRNA and protein levels of knockdown ITGB1. All data are expressed as mean ± SD (*n* = 3) and *p < 0.05, **p < 0.01, ***p < 0.001 compared with si‐NC group.

## References

[1] M. Izquierdo , R. A. Merchant , J. E. Morley , et al., “International Exercise Recommendations in Older Adults (ICFSR): Expert Consensus Guidelines,” Journal of Nutrition, Health & Aging 25 (2021): 824–853, 10.1007/s12603-021-1665-8. PMC12369211 34409961

[2] K. Pacholek and M. Sobieszczanska , “Sarcopenia Identification During Comprehensive Geriatric Assessment,” International Journal of Environmental Research and Public Health 19 (2021): 32, 10.3390/ijerph19010032. 35010295 PMC8751172

[3] L. K. Chen , L. K. Liu , J. Woo , et al., “Sarcopenia in Asia: Consensus Report of the Asian Working Group for Sarcopenia,” Journal of the American Medical Directors Association 15 (2014): 95–101, 10.1016/j.jamda.2013.11.025. 24461239

[4] A. J. Cruz‐Jentoft , J. P. Baeyens , J. M. Bauer , et al., “Sarcopenia: European Consensus on Definition and Diagnosis: Report of the European Working Group on Sarcopenia in Older People,” Age and Ageing 39 (2010): 412–423, 10.1093/ageing/afq034. 20392703 PMC2886201

[5] J. M. Petrosino , A. Leask , and F. Accornero , “Genetic Manipulation of CCN2/CTGF Unveils Cell‐Specific ECM‐Remodeling Effects in Injured Skeletal Muscle,” FASEB Journal 33 (2019): 2047–2057, 10.1096/fj.201800622RR. 30216109 PMC6338641

[6] P. Sosa , E. Alcalde‐Estevez , A. Asenjo‐Bueno , et al., “Aging‐Related Hyperphosphatemia Impairs Myogenic Differentiation and Enhances Fibrosis in Skeletal Muscle,” Journal of Cachexia, Sarcopenia and Muscle 12 (2021): 1266–1279, 10.1002/jcsm.12750. 34337906 PMC8517361

[7] Y. Wu , Y. Wu , Y. Yang , et al., “Lysyl Oxidase‐Like 2 Inhibitor Rescues D‐Galactose–Induced Skeletal Muscle Fibrosis,” Aging Cell 21 (2022): e13659, 10.1111/acel.13659. 35712918 PMC9282848

[8] A. S. Brack , M. J. Conboy , S. Roy , et al., “Increased Wnt Signaling During Aging Alters Muscle Stem Cell Fate and Increases Fibrosis,” Science 317 (2007): 807–810, 10.1126/science.1144090. 17690295

[9] A. K. Lyu , S. Y. Zhu , J. L. Chen , et al., “Inhibition of TLR9 Attenuates Skeletal Muscle Fibrosis in Aged Sarcopenic Mice via the p53/SIRT1 Pathway,” Experimental Gerontology 122 (2019): 25–33, 10.1016/j.exger.2019.04.008. 31003004

[10] R. K. Assoian and M. A. Schwartz , “Coordinate Signaling by Integrins and Receptor Tyrosine Kinases in the Regulation of G1 Phase Cell‐Cycle Progression,” Current Opinion in Genetics & Development 11 (2001): 48–53, 10.1016/s0959-437x(00)00155-6. 11163150

[11] B. L. Justo and M. G. Jasiulionis , “Characteristics of TIMP1, CD63, and Beta1‐Integrin and the Functional Impact of Their Interaction in Cancer,” International Journal of Molecular Sciences 22 (2021): 9319, 10.3390/ijms22179319. 34502227 PMC8431149

[12] L. Sun , S. Guo , Y. Xie , and Y. Yao , “The Characteristics and the Multiple Functions of Integrin Beta1 in Human Cancers,” Journal of Translational Medicine 21 (2023): 787, 10.1186/s12967-023-04696-1. 37932738 PMC10629185

[13] D. Gullberg , T. Velling , L. Lohikangas , and C. F. Tiger , “Integrins During Muscle Development and in Muscular Dystrophies,” Frontiers in Bioscience 3 (1998): D1039–D1050, 10.2741/a344. 9778539

[14] S. C. Schuler , J. M. Kirkpatrick , M. Schmidt , et al., “Extensive Remodeling of the Extracellular Matrix During Aging Contributes to Age‐Dependent Impairments of Muscle Stem Cell Functionality,” Cell Reports 35 (2021): 109223, 10.1016/j.celrep.2021.109223. 34107247

[15] W. Luo , Z. Lin , J. Chen , et al., “TMEM182 Interacts With Integrin Beta 1 and Regulates Myoblast Differentiation and Muscle Regeneration,” Journal of Cachexia, Sarcopenia and Muscle 12 (2021): 1704–1723, 10.1002/jcsm.12767. 34427057 PMC8718073

[16] X. Shen , J. Tang , R. Jiang , et al., “CircRILPL1 Promotes Muscle Proliferation and Differentiation via Binding miR‐145 to Activate IGF1R/PI3K/AKT Pathway,” Cell Death & Disease 12 (2021): 142, 10.1038/s41419-021-03419-y. 33542215 PMC7862392

[17] H. Li , W. Xu , Y. Ma , S. Zhou , and R. Xiao , “Milk Fat Globule Membrane Protein Promotes C(2)C(12) Cell Proliferation Through the PI3K/Akt Signaling Pathway,” International Journal of Biological Macromolecules 114 (2018): 1305–1314, 10.1016/j.ijbiomac.2018.04.026. 29634969

[18] D. A. Goncalves , W. A. Silveira , L. H. Manfredi , et al., “Insulin/IGF1 Signalling Mediates the Effects of Beta(2) ‐Adrenergic Agonist on muScle Proteostasis and Growth,” Journal of Cachexia, Sarcopenia and Muscle 10 (2019): 455–475, 10.1002/jcsm.12395. 30932373 PMC6463755

[19] S. Schiaffino and C. Mammucari , “Regulation of Skeletal Muscle Growth by the IGF1‐Akt/PKB Pathway: Insights From Genetic Models,” Skeletal Muscle 1 (2011): 4, 10.1186/2044-5040-1-4. 21798082 PMC3143906

[20] M. Wang , R. Hu , Y. Wang , et al., “Atractylenolide III Attenuates Muscle Wasting in Chronic Kidney Disease via the Oxidative Stress–Mediated PI3K/AKT/mTOR Pathway,” Oxidative Medicine and Cellular Longevity 2019 (2019): 1875471, 10.1155/2019/1875471. 31178951 PMC6501186

[21] T. Kanzleiter , M. Rath , S. W. Gorgens , et al., “The Myokine Decorin is Regulated by Contraction and Involved in Muscle Hypertrophy,” Biochemical and Biophysical Research Communications 450 (2014): 1089–1094, 10.1016/j.bbrc.2014.06.123. 24996176

[22] E. Kubo , S. Shibata , T. Shibata , H. Sasaki , and D. P. Singh , “Role of Decorin in the Lens and Ocular Diseases,” Cells 12 (2022): 74, 10.3390/cells12010074. 36611867 PMC9818407

[23] Y. Jia , Q. Feng , B. Tang , et al., “Decorin Suppresses Invasion and EMT Phenotype of Glioma by Inducing Autophagy via c‐Met/Akt/mTOR Axis,” Frontiers in Oncology 11 (2021): 659353, 10.3389/fonc.2021.659353. 34386415 PMC8353327

[24] S. Gupta , F. Buyank , N. R. Sinha , et al., “Decorin Regulates Collagen Fibrillogenesis During Corneal Wound Healing in Mouse In Vivo,” Experimental Eye Research 216 (2022): 108933, 10.1016/j.exer.2022.108933. 35031282 PMC8885890

[25] P. Kaur , N. Verma , P. Garg , et al., “Myokines Are Associated With Progression, Course and Mortality in Alcohol‐Associated Liver Disease,” Alimentary Pharmacology & Therapeutics 60 (2024): 1005–1020, 10.1111/apt.18202. 39135311

[26] F. Cianfarani , E. De Domenico , A. Nystrom , et al., “Decorin Counteracts Disease Progression in Mice With Recessive Dystrophic Epidermolysis Bullosa,” Matrix Biology 81 (2019): 3–16, 10.1016/j.matbio.2018.12.001. 30528862

[27] K. G. Danielson , H. Baribault , D. F. Holmes , H. Graham , K. E. Kadler , and R. V. Iozzo , “Targeted Disruption of Decorin Leads to Abnormal Collagen Fibril Morphology and Skin Fragility,” Journal of Cell Biology 136 (1997): 729–743, 10.1083/jcb.136.3.729. 9024701 PMC2134287

[28] L. Mao , J. Yang , J. Yue , et al., “Decorin Deficiency Promotes Epithelial–Mesenchymal Transition and Colon Cancer Metastasis,” Matrix Biology 95 (2021): 1–14, 10.1016/j.matbio.2020.10.001. 33065248 PMC7870527

[29] Y. Wu , Y. Wu , J. Yu , et al., “Irisin Ameliorates D‐Galactose–Induced Skeletal Muscle Fibrosis via the PI3K/Akt Pathway,” European Journal of Pharmacology 939 (2023): 175476, 10.1016/j.ejphar.2022.175476. 36539073

[30] R. Srikuea and M. Hirunsai , “TGF‐Beta1 Stimulation and VDR‐Dependent Activation Modulate Calcitriol Action on Skeletal Muscle Fibroblasts and Smad Signalling‐Associated Fibrogenesis,” Scientific Reports 13 (2023): 13811, 10.1038/s41598-023-40978-w. 37612333 PMC10447566

[31] Y. Wu , Y. Wu , J. Yu , et al., “Irisin Alters D‐Galactose–Induced Apoptosis by Increasing Caveolin‐1 Expression in C2C12 Myoblasts and Skeletal Muscle Fibroblasts,” Molecular and Cellular Biochemistry 480 (2025): 577–588, 10.1007/s11010-024-04990-6. 38581552

[32] M. Rozo , L. Li , and C. M. Fan , “Targeting Beta1‐Integrin Signaling Enhances Regeneration in Aged and Dystrophic Muscle in Mice,” Nature Medicine 22 (2016): 889–896, 10.1038/nm.4116. PMC4974124 27376575

[33] P. Wiedmer , T. Jung , J. P. Castro , et al., “Sarcopenia—Molecular Mechanisms and Open Questions,” Ageing Research Reviews 65 (2021): 101200, 10.1016/j.arr.2020.101200. 33130247

[34] H. Zhang , X. Wu , J. Liang , M. Kirberger , and N. Chen , “Irisin, an Exercise‐Induced Bioactive Peptide Beneficial for Health Promotion During Aging Process,” Ageing Research Reviews 80 (2022): 101680, 10.1016/j.arr.2022.101680. 35793739

[35] B. P. Carson , “The Potential Role of Contraction‐Induced Myokines in the Regulation of Metabolic Function for the Prevention and Treatment of Type 2 Diabetes,” Frontiers in Endocrinology (Lausanne) 8 (2017): 97, 10.3389/fendo.2017.00097. PMC5411437 28512448

[36] B. K. Koo , S. H. Um , D. S. Seo , et al., “Growth Differentiation Factor 15 Predicts Advanced Fibrosis in Biopsy‐Proven Non‐Alcoholic Fatty Liver Disease,” Liver International 38 (2018): 695–705, 10.1111/liv.13587. 28898507

[37] M. Kumari , A. Mohan , C. M. Ecelbarger , A. Gupta , N. Prasad , and S. Tiwari , “miR‐451 Loaded Exosomes Are Released by the Renal Cells in Response to Injury and Associated With Reduced Kidney Function in Human,” Frontiers in Physiology 11 (2020): 234, 10.3389/fphys.2020.00234. 32322216 PMC7158952

[38] H. H. Wang , Y. Zhang , T. Q. Qu , et al., “Nobiletin Improves D‐Galactose–Induced Aging Mice Skeletal Muscle Atrophy by Regulating Protein Homeostasis,” Nutrients 15 (2023): 1801, 10.3390/nu15081801. 37111020 PMC10146842

[39] S. Tian , H. Zhao , H. Guo , W. Feng , C. Jiang , and Y. Jiang , “Propolis Ethanolic Extract Attenuates D‐Gal–Induced C2C12 Cell Injury by Modulating Nrf2/HO‐1 and p38/p53 Signaling Pathways,” International Journal of Molecular Sciences 24 (2023): 6408, 10.3390/ijms24076408. 37047379 PMC10094417

[40] Y. F. Yang , W. Yang , Z. Y. Liao , et al., “MICU3 Regulates Mitochondrial Ca(2+)‐Dependent Antioxidant Response in Skeletal Muscle Aging,” Cell Death & Disease 12 (2021): 1115, 10.1038/s41419-021-04400-5. 34845191 PMC8630021

[41] X. Zheng , P. Wang , L. Li , et al., “Cancer‐Associated Fibroblasts Promote Vascular Invasion of Hepatocellular Carcinoma via Downregulating Decorin–Integrin Beta1 Signaling,” Frontiers in Cell and Development Biology 9 (2021): 678670, 10.3389/fcell.2021.678670. PMC8421641 34504839

[42] S. P. Lall , Z. W. Alsafwani , S. K. Batra , and P. Seshacharyulu , “ASPORIN: A Root of the Matter in Tumors and Their Host Environment,” Biochimica Et Biophysica Acta. Reviews on Cancer 1879 (2024): 189029, 10.1016/j.bbcan.2023.189029. 38008263 PMC10872503

[43] A. Martin , Y. S. Gallot , and D. Freyssenet , “Molecular Mechanisms of Cancer Cachexia‐Related Loss of Skeletal Muscle Mass: Data Analysis From Preclinical and Clinical Studies,” Journal of Cachexia, Sarcopenia and Muscle 14 (2023): 1150–1167, 10.1002/jcsm.13073. 36864755 PMC10235899

[44] M. Serova , B. Didry‐Barca , R. Deloux , et al., “BIO101 Stimulates Myoblast Differentiation and Improves Muscle Function in Adult and Old Mice,” Journal of Cachexia, Sarcopenia and Muscle 15 (2024): 55–66, 10.1002/jcsm.13326. 38064183 PMC10834314

[45] R. He , J. Peng , P. Yuan , F. Xu , and W. Wei , “Divergent Roles of BECN1 in LC3 Lipidation and Autophagosomal Function,” Autophagy 11 (2015): 740–747, 10.1080/15548627.2015.1034404. 25955014 PMC4509441

