# Supplementary material for: Decorin Deficiency Promotes D‐Galactose–Induced Skeletal Muscle Atrophy and Fibrosis by Regulating ITGB1/Akt/mTOR Signalling Pathway
Source: J Cachexia Sarcopenia Muscle. 2025 Dec 5;16(6):e70144. doi: 10.1002/jcsm.70144 (PMC12680447; doi:10.1002/jcsm.70144)
Supplement: Supplementary file 1 — Table S1: The sequences of siRNAs used for RNA interference and control. Table S2: Sequences of primers used for PCR or qRT‐PCR amplification. Table S3: Primary antibodies were utilised for Western blotting, immunofluorescence and co‐immunoprecipitation. Figure S1: si‐Dcn exacerbated autophagy in D‐gal–induced NOR‐10 cells. (a) Effect of D‐gal (50–250 mM) on the proliferation of NOR‐10 cells. (b) Effect of different doses of D‐gal treatment on NOR‐10 cells viability at different periods. (c) Western blotting analysed the protein levels of decorin and β‐actin in D‐gal–induced NOR‐10 cells (n = 3). (d) The mRNA and protein levels of Decorin after knockdown Dcn in NOR‐10 cells (n = 3). (e) Protein levels of LC3b and p62 in D‐gal–induced NOR‐10 cells of knockdown Dcn. (f) The relative protein levels of the target proteins were normalised to those of β‐actin. All data are expressed as mean ± SD (n = 6) and *p < 0.05, **p < 0.01, ***p < 0.001 compared with the Ctrl and si‐NC groups. Figure S2: Decorin deficiency exacerbated skeletal muscle wasting in D‐gal–induced mice. (a) The genotype of Dcn was identified by PCR analysis. (b) The changes body weight of Dcn+/+ mice with saline and Dcn+/+ and Dcn−/− mice with D‐gal–induced groups (n = 6). (c) The body weight of Dcn+/+, Dcn+/+–D‐gal and Dcn−/−–D‐gal groups in 10 weeks. (d) The weights of quadriceps femoris (Qu), gastrocnemius (Gast) and tibialis anterior (TA) in Dcn+/+, Dcn+/+–D‐gal or Dcn−/−–D‐gal groups. (e) Relative mRNA levels of atrophic genes in Gast muscles. (f) Relative mRNA levels of age gene in Gast muscles. (g) Relative mRNA levels of fibrosis genes in Gast muscles. (h) Western blotting assays the protein levels of myogenin and MyoD1 in Gast muscles. (i) The relative protein levels of the target proteins were normalised to those of GAPDH. All data are expressed as mean ± SD (n = 6) and *p < 0.05, **p < 0.01, ***p < 0.001 compared with Dcn+/+ group. Figure S3: Dcn overexpression reversed D‐gal–induced NOR‐10 cel [file JCSM-16-e70144-s001.docx]

**Supplementary Material**

**1. Materials and methods**

**Table S1** The sequences of siRNAs used for RNA interference and control

| **Gene name** | **Sequence (5’-3’)** |
| --- | --- |
| si-Dcn-631 | CCUGUCUAAGAACCAACUAAA (dT) |
|  | UUUAGUUGGUUCUUAGACAGG (dT) |
| si-Dcn-730 | CGACUUCAAUGGACUGAACAA (dT) |
|  | UUGUUCAGUCCAUUGAAGUCG (dT) |
| si-Dcn-945 | DCCUGAAAGGACUGAUUAAUU (dT) |
|  | AAUUAAUCAGUCCUUUCAGGC (dT) |
| si-ITGB1-490 | GCACGAUGUGAUGAUUUAGAA (dT) |
|  | UUCUAAAUCAUCACAUCGUGC (dT) |
| si-ITGB1-1238 | GCCAUUACUAUGAUUAUCCUU (dT) |
|  | AAGGAUAAUCAUAGUAAUGGC (dT) |
| si-ITGB1-2174 | CCAAGUUUCAAGGGCCAACUU (dT) |
|  | AAGUUGGCCCUUGAAACUUGG (dT) |
| si-NC | UUCUCCGAACGUGUCACGUTT (dT)  ACGUGACACGUUCGGAGAATT (dT) |

**Table S2** Sequences of primers used for PCR or qRT-PCR amplification

| **Genes** | **Position** | **Sequences** | **Size（bp）** | **Application** |
| --- | --- | --- | --- | --- |
| mDcn | Forward | CCTTCTGGCACAAGTCTCTTGG | 161 | PCR |
|  | Reverse | TCGAAGATGACACTGGCATCGG |  |  |
|  | PGK-1 | TGGATGTGGAATGTGTGCGAGG |  |  |
| mDcn | Forward | AGATCACCAAGCTGCGGAAA | 105 | qRT-PCR |
|  | Reverse | AGGCTCCGTTTTCAATCCCA |  |  |
| mFN | Forward | ATGTGGACCCCTCCTGATAGT | 124 | qRT-PCR |
|  | Reverse | GCCCAGTGATTTCAGCAAAGG |  |  |
| mActa2 | Forward | CCCAGACATCAGGGAGTAATGG | 104 | qRT-PCR |
|  | Reverse | TCTATCGGATACTTCAGCGTCA |  |  |
| mCol3a1 | Forward | CTGTAACATGGAAACTGGGGAAA | 144 | qRT-PCR |
|  | Reverse | CCATAGCTGAACTGAAAACCACC |  |  |
| mMSTN | Forward | CAGCCTGAATCCAACTTAGG | 167 | qRT-PCR |
|  | Reverse | TCGCAGTCAAGCCCAAAGTC |  |  |
| mMuRF-1 | Forward | GTGTGAGGTGCCTACTTGCTC | 101 | qRT-PCR |
|  | Reverse | GCTCAGTCTTCTGTCCTTGGA |  |  |
| mAtrogin-1 | Forward | CTCAGTGAGGACCGGCTACT | 103 | qRT-PCR |
|  | Reverse | AATCCAGCTGCCCTTTGTCA |  |  |
| mTrp53 | Forward | CTCCCCAGCATCTTATCCGG | 102 | qRT-PCR |
|  | Reverse | CGGGTGGCTCATAAGGTACC |  |  |
| mCdkn2a | Forward | CGGGGACATCAAGACATCGT | 114 | qRT-PCR |
|  | Reverse | GAAGCTATGCCCGTCGGTC |  |  |
| mIL-6 | Forward | TAGTCCTTCCTACCCCAATTTCC | 76 | qRT-PCR |
|  | Reverse | TTGGTCCTTAGCCACTCCTTC |  |  |
| mIL-1β | Forward | CCAGCTTCAAATCTCGCAGC | 104 | qRT-PCR |
|  | Reverse | ACGGGAAAGACACAGGTAGC |  |  |
| **mTNF-α** | **Forward** | **ATGGCCTCCCTCTCATCAGT** | **97** | **qRT-PCR** |
|  | **Reverse** | **TTTGCTACGACGTGGGCTAC** |  |  |
| RPL35A | Forward | CCGAGATGAAACGGAGTTCTAC | 120 | qRT-PCR |
|  | Reverse | GTTACCTTTCCCCAGATCACT |  |  |

**Table S3** Primary antibodies were utilized for Western blotting, immunofluorescence, and Co-Immunoprecipitation

| **Antibody** | **Company (Lot.)** | **Working dilutions** |
| --- | --- | --- |
| Decorin | Proteintech, 14667-1-AP | WB: 1:2000, IF: 1:100 |
| Decorin | Abcam, ab175404 | CO-IP: 3 μg |
| α-SMA | Abcam, ab5694 | WB: 1:2000 |
| α-SMA | Genetex, GTX100034 | IF: 1:50 |
| MuRF-1 | Proteintech, 55456-1-AP | WB: 1:5000 |
| Atrogin-1 | Proteintech, 67172-1-Ig | WB: 1:5000 |
| p16INK4a | Wanleibio, WL01418 | WB: 1:1000 |
| p53 | Proteintech, 80077-1-RR | WB: 1:1000 |
| Fibronectin | Genetex, GTX112794 | WB: 1:1000 |
| COL1 | Genetex, GTX26308 | WB: 1:1000 |
| ITGB1 | Wanleibio, WL01615 | WB: 1:1000 |
| ITGB1 | Santa Cruz, sc-53711 | CO-IP: 3 μg, IF: 1:50 |
| p-S473-Akt | Cell Signaling Technology, #13038T | WB: 1:1000 |
| Akt | Proteintech, 60203-2-Ig | WB: 1:1000 |
| p-p70 S6K | Cell Signaling Technology, #9205 | WB: 1:1000 |
| p70 | Proteintech, 14485-1-AP | WB: 1:1000 |
| p-Ser2448-mTOR | Proteintech, 67778-1-Ig | WB: 1:1000 |
| mTOR | Cell Signaling Technology, #2983 | WB: 1:1000 |
| Phospho-4E-BP1 | Cell Signaling Technology, #2855 | WB: 1:1000 |
| **NLRP3** | **Proteintech, 68102-1** | **WB: 1:2000** |
| **p-p65** | **Cell Signaling Technology, #3033** | **WB: 1:1000** |
| **p65** | **Cell Signaling Technology, #4764** | **WB: 1:1000** |
| **p62** | **Affinity, AF5384** | **WB: 1:1000** |
| **LC3b** | **Abcepta, APG8B** | **WB: 1:1000** |
| **MYOG** | **Affinity, DF8273** | **WB: 1:1000** |
| **MyoD1** | **Wanleibio, WL04662** | **WB: 1:1000** |
| **p21** | **Abways, CY5543** | **WB: 1:1000** |
| **Puromycin** | **Bioworld, BS78130** | **WB: 1:3000** |
| **PAX7** | **Affinity, AF7584** | **WB: 1:1000** |
| β-actin | Servicebio, ZB15001-HRP-100 | WB: 1:3000 |
| GAPDH | Servicebio, ZB15004-HRP-100 | WB: 1:3000 |

**2. Supplementary figures**

**Figure S1** **si-Dcn exacerbated autophagy in D-gal-induced NOR-10 cells.** (a) Effect of D-gal (50-250 mM) on the proliferation of NOR-10 cells. (b) Effect of different doses of D-gal treatment on NOR-10 cells viability at different periods. **(c) Western blotting analyzed the protein levels of Decorin and β-actin in D-gal-induced NOR-10 cells (n=3). (d) The mRNA and protein levels of Decorin after knockdown Dcn in NOR-10 cells (n=3).** **(e) Protein levels of LC3b and p62 in D-gal-induced NOR-10 cells of knockdown Dcn. (f) The relative protein levels of the target proteins were normalized to those of β-actin.** All data are expressed as mean ± SD (n=6) and **p* < 0.05, ***p* < 0.01, ****p* < 0.001 compared with Ctrl and si-NC groups.

**
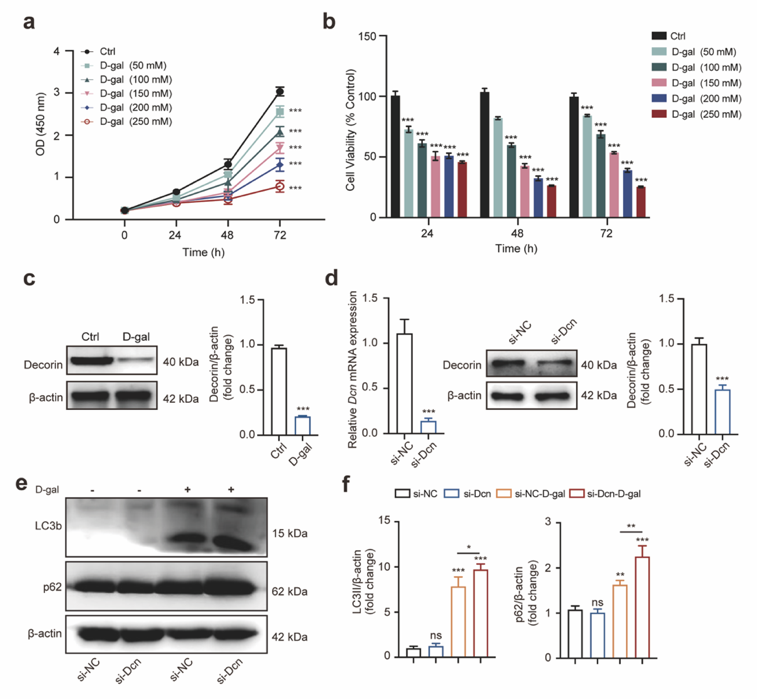
**

**Figure S2** Decorin deficiency exacerbated skeletal muscle wasting in D-gal-induced mice. (a) The genotype of Dcn was identified by PCR analysis. (b) The changes body weight of Dcn^+/+^ mice with saline and Dcn^+/+^ and Dcn^-/-^ mice with D-gal-induced groups **(n=6)**. (c) The body weight of Dcn^+/+^, Dcn^+/+^-D-gal and Dcn^-/-^-D-gal groups in 10 weeks. (d) The weights of quadriceps femoris (Qu), gastrocnemius (Gast) and tibialis anterior (TA) in Dcn^+/+^, Dcn^+/+^-D-gal or Dcn^-/-^-D-gal groups; (e) Relative mRNA levels of atrophic genes in Gast muscles; (f) Relative mRNA levels of age gene in Gast muscles; (g) Relative mRNA levels of fibrosis genes in Gast muscles; **(h) Western blotting assays the protein levels of Myogenin and MyoD1 in Gast muscles. (i) The relative protein levels of the target proteins were normalized to those of GAPDH.** All data are expressed as mean ± SD (n=6) and **p* < 0.05, ***p* < 0.01, ****p* < 0.001 compared with Dcn^+/+^ group.

**
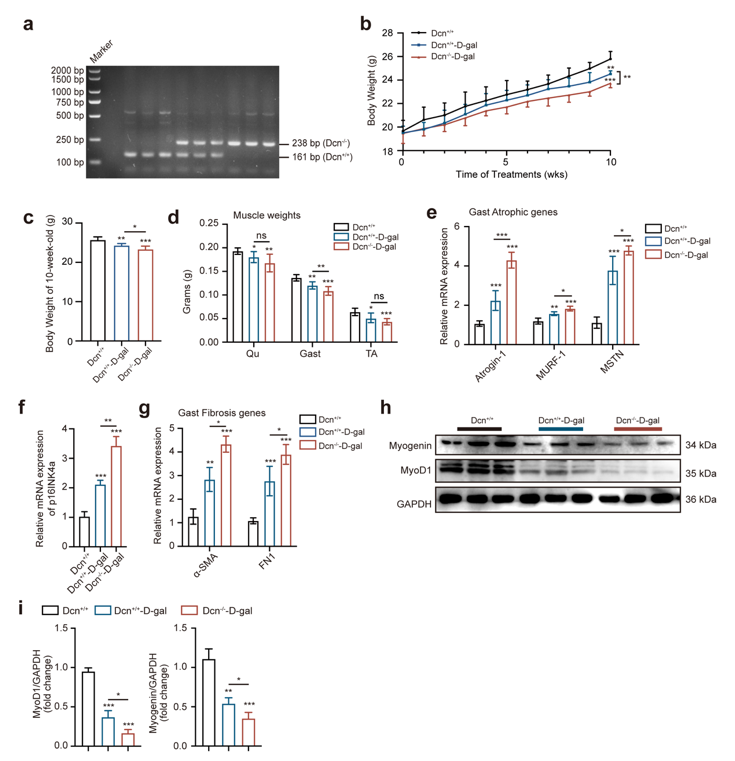
**

**Figure S3 Dcn over-expression reversed D-gal-induced NOR-10 cells autophagy.** **(a) Quantification of overexpression efficiency of Dcn in NOR-10 cells by qRT-PCR (n=3). (b) Protein levels of Decorin in NC and Dcn^OE^ groups by Western blotting (n=3). (c) Western blotting analyzed the protein levels of puromycin in NC, Dcn^OE^, NC-D-gal and Dcn^OE^-D-gal groups. (d) The relative protein levels of the target proteins were normalized to those of β-actin. (e) Western blotting analyzed the protein levels of p62 and LC3b in NC, Dcn^OE^, NC-D-gal and Dcn^OE^-D-gal groups. (f) The relative protein levels of the target proteins were normalized to those of β-actin. All data are expressed as mean ± SD (n=6) and **p* < 0.05, ***p* < 0.01, ****p* < 0.001 compared with NC group.**

**
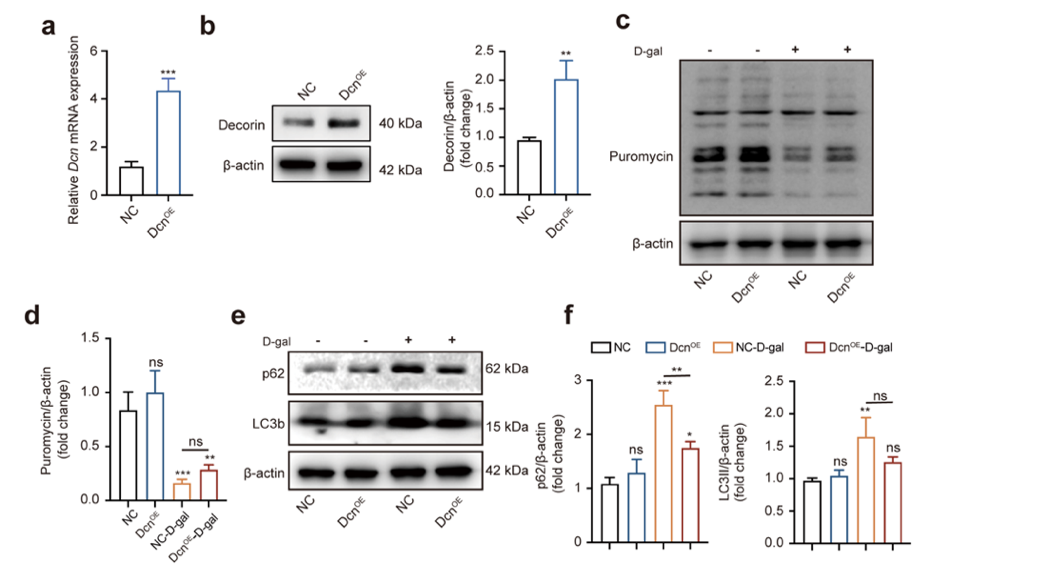
**

**Figure S4 Recombinant** Decorin reduced **autophagy in D-gal-induced NOR-10 cells.** (a) Effect of 100 mM D-gal and Decorin (5-80 ng/ml) on the proliferation of NOR-10 cells. (b) Effect of different doses of Decorin treatment on NOR-10 cell viability at different periods. (c) SA-β-Gal staining and quantification in Ctrl, D-gal and D-gal+Decorin groups **(n=3)**. (d) The mRNA levels of atrophic, fibrosis and ageing genes in Ctrl, D-gal and D-gal+Decorin groups were measured by qRT-PCR analysis **(n=3)**. (e) Immunofluorescence staining of α-SMA in Ctrl, D-gal and D-gal+Decorin groups. **(f) The corresponding statistical analysis. (g) Western blotting analyzed the protein levels of p62 and LC3b in Ctrl, D-gal and D-gal+Decorin groups. (h, i) The relative protein levels of the target proteins were normalized to those of β-actin. (j, k) Relative mRNA levels of aging genes of primary skeletal muscle cells in Ctrl, D-gal and D-gal+Decorin groups.** Scale bar, 100 μm. All data are expressed as mean ± SD (n=3) and **p* < 0.05, ***p* < 0.01, ****p* < 0.001 compared with Ctrl group.

**
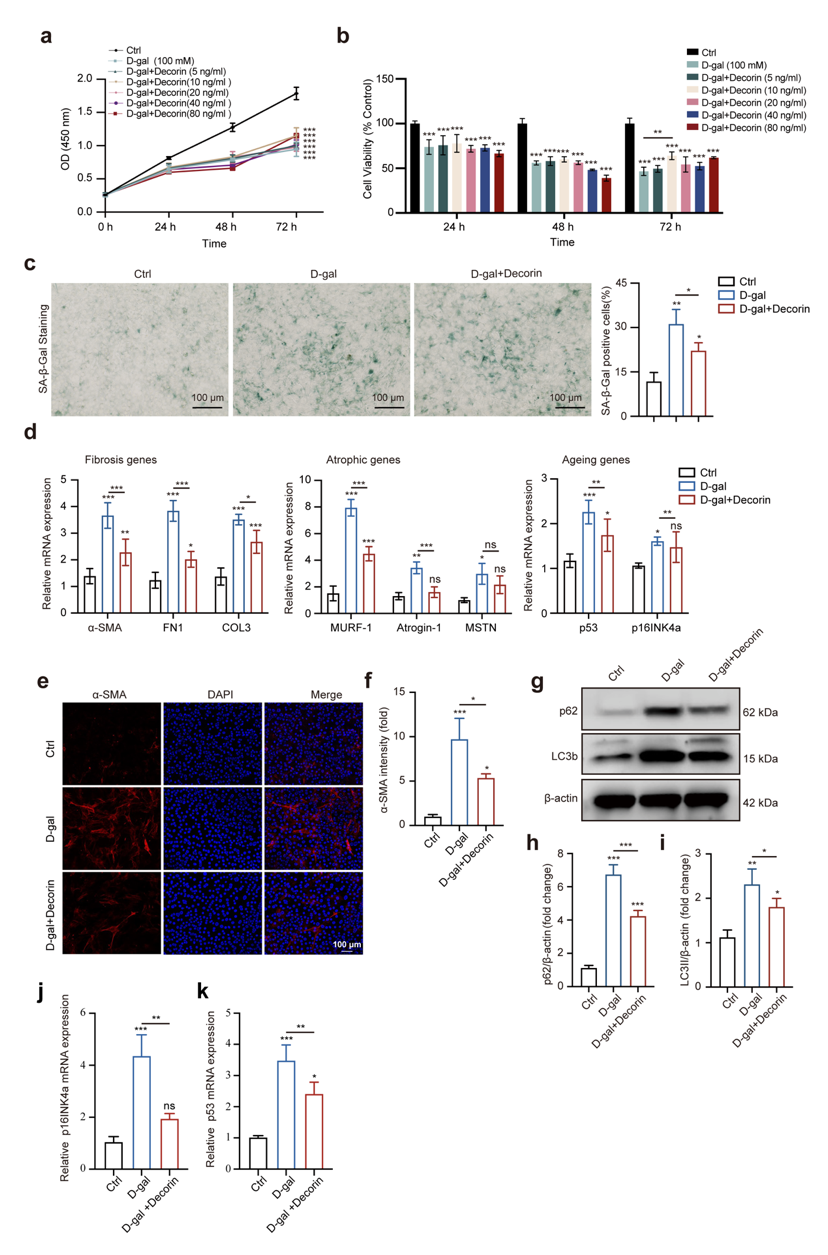
**

**Figure S5** Decorin deficiency reduced the expression of ITGB1 in D-gal-induced aged mice. (a) Western blotting assays the protein levels of ITGB1, GAPDH and the corresponding statistical analysis in Dcn^+/+^, Dcn^+/+^-D-gal or Dcn^-/-^-D-gal groups. All data are expressed as mean ± SD (n=3) and **p* < 0.05, ***p* < 0.01, ****p* < 0.001 compared with Dcn^+/+^ group.

**
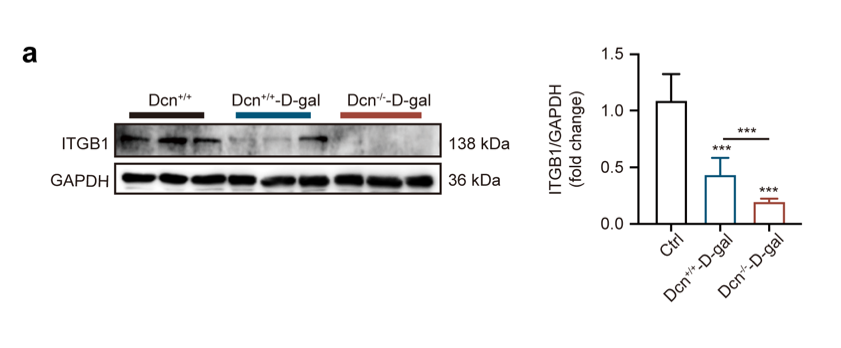
**

**Figure S6** The knockdown efficiency of siRNAs against ITGB1 in NOR-10 cells with over-expression of Dcn. (a-b) The mRNA and protein levels of knockdown ITGB1. All data are expressed as mean ± SD (n=3) and **p* < 0.05, ***p* < 0.01, ****p* < 0.001 compared with si-NC group.

**
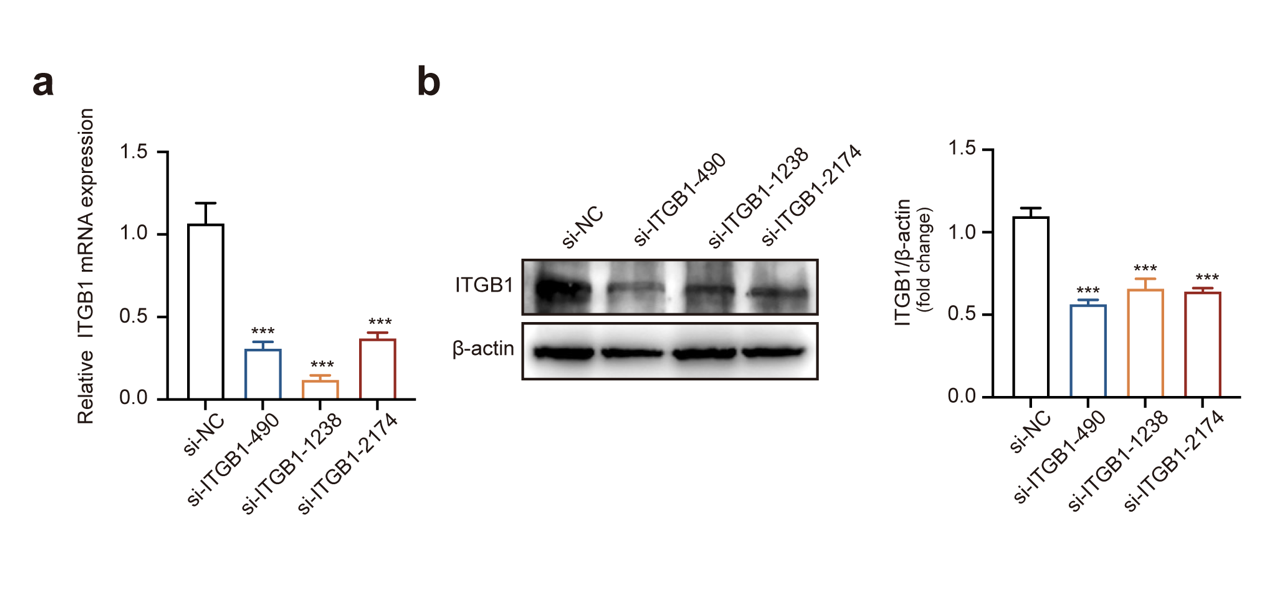
**
